# Synthesis of Hafnium(IV) Polyaminoacetates

**DOI:** 10.3390/molecules26123725

**Published:** 2021-06-18

**Authors:** Alexandra T. Shulyak, Evgeniy O. Bortnikov, Alexey S. Kubasov, Nikita A. Selivanov, Alexey A. Lipengolts, Andrey P. Zhdanov, Alexander Yu. Bykov, Konstantin Yu. Zhizhin, Nikolai T. Kuznetsov

**Affiliations:** 1 Kurnakov Institute of General and Inorganic Chemistry, Russian Academy of Sciences, Leninskiy pr. 31, 119991 Moscow, Russia; fobosax@mail.ru (A.S.K.); govee@yandex.ru (N.A.S.); zhdanov@igic.ras.ru (A.P.Z.); bykov@igic.ras.ru (A.Y.B.); zhizhin@igic.ras.ru (K.Y.Z.); ntkuz@igic.ras.ru (N.T.K.); 2 Inorganic Chemistry Department, Lomonosov Institute of Fine Chemical Technologies, MIREA-Russian Technological University, pr. Vernadskogo, 86, 119454 Moscow, Russia; 3 Organic Chemistry Department, Weizmann Institute of Science, 234 Herzl Street, Rehovot 7610001, Israel; bortnikovevol@gmail.com; 4 N.N. Blokhin National Medical Research Center of Oncology, Federal State Budgetary Institution, Ministry of Health of the Russian Federation (N.N. Blokhin NMRCO), Kashirskoye Shosse 24, 115478 Moscow, Russia; lipengolz@gmail.com

**Keywords:** hafnium, complexonates, coordination chemistry, DTPA, contrast agent, structure elucidation, ligand effect

## Abstract

The interaction of hafnium(IV) salts (oxide-dichloride, chloride, and bromide) with nitrilotriacetic acid (NTA), diethylenetriamminepentaacetic acid (DTPA), 1,2-diaminocyclohexanetetraacetic acid (CDTA), 1,3-dipropylmino-2-hydroxy *N*,*N*,*N*′,*N*′-tetraacetic acid (dpta), and *N*-(2-hydroxyethyl)ethylenediamine triacetic acid (HEDTA) has been studied. The corresponding complexes Na_2_[Hf(NTA)_2_]·3H_2_O (**1**), Na[HfDTPA]·3H_2_O (**2**), [HfCDTA(H_2_O)_2_] (**3**), and Na[Hf_2_(dpta)_2_]·7.5H_2_O·0.5C_2_H_5_OH (**4**) have been isolated and characterized and their structures have been determined by single crystal X-ray diffraction. Biological studies of [HfCDTA(H_2_O)_2_] have shown that in 5% glucose solution this complex has low toxicity and good contrasting ability.

## 1. Introduction

**Figure 1:**
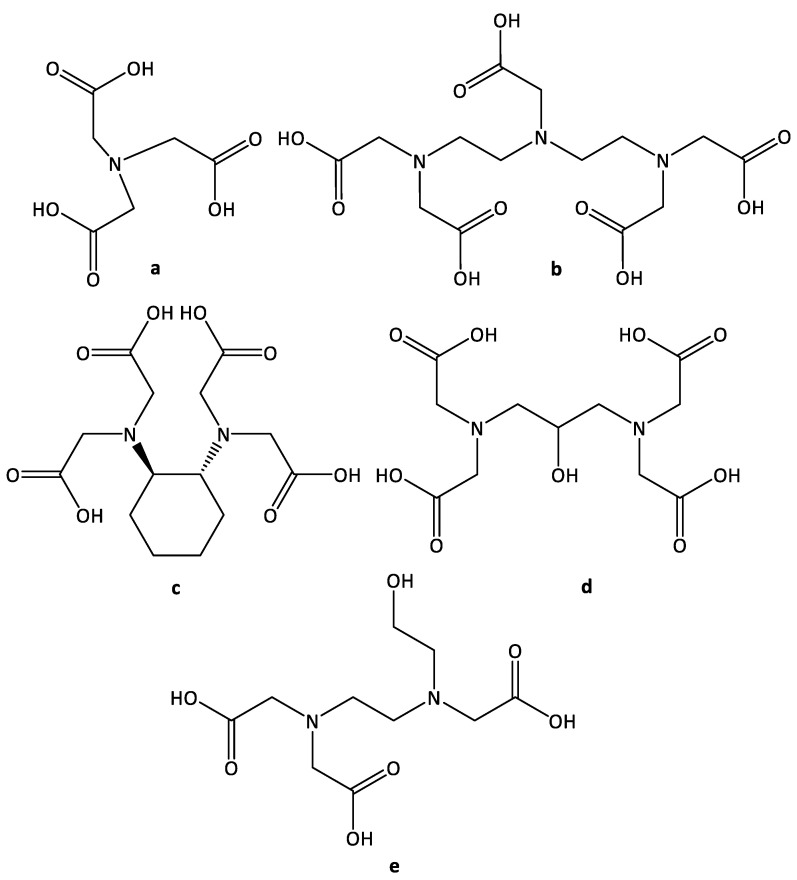
The structure of the ligands: (**a**) nitrilotriacetic acid (NTA); (**b**) diethylenetriamminepentaacetic acid (DTPA), (**c**) 1,2-diaminocyclohexanetetraacetic acid (CDTA), (**d**) 1,3-dipropylmino-2-hydroxy *N*,*N*,*N*′,*N*′-tetraacetic acid (dpta), (**e**) *N*-(2-hydroxyethyl)ethylenediamine triacetic acid (HEDTA).

Currently, radiological research methods are the main methods for diagnosing malignant neoplasms. To obtain better images, contrast agents are used [1]. For example, complexes of gadolinium(III) with polyaminocarboxylic acids (for example, diethylenetriamine pentaacetic acid (DTPA)) (Figure 1) are successfully used for magnetic resonance imaging [2,3,4]. Due to the presence of several donor atoms, such acids have a strong chelating effect and can form stable complexes with heavy element atoms, which allows them to be safely introduced and removed from the body [5,6].

At the same time, only organoiodine compounds are mainly used for computed tomography [7,8,9], and attempts to use gadolinium preparations for this method were unsuccessful [9]. Iodine has a sufficiently large radius, which contributes to a high absorption coefficient of X-ray radiation [10]; however, in [11], water-soluble complexes containing hafnium(IV) were obtained, which proved to be better as a contrast agent in comparison with the preparation Iopromide©.

In this regard, it seems possible to synthesize contrast agents based on hafnium, namely, based on its complexes with polyaminocarboxylic acids [12]. Despite the similarity of the values of the atomic radii, only the isostructural complexes of zirconium have been mainly studied [13,14]. The chemistry of hafnium complexes has been scarcely studied because of their higher tendency to hydrolysis and because in practice hafnium complexes are not isolated from solutions in pure form. Nevertheless, the structures of potassium and guanidinium salts of Hf-DTPA were reported [15,16].

Hydrolysis of hafnium in a medium with a high pH value [17] complicates the preparation and identification of complexes. Based on the literature data [14,18,19,20,21,22], in the course of this work it was possible to establish the optimal conditions for the preparation and isolation of complexes in a high yield.

Thus, the aim of this work is to improve the existing methods for the synthesis of hafnium complexes with polyamino carboxylic acids and to isolate them from solution for biological studies.

## 2. Results and Discussion 

### 2.1. Synthesis

The complexes **1**–**5** were synthesized based on the previously cited literature data [12,16,23]. In the case of acids DTPA, CDTA, DPTA, HEDTA, the ratio of metal:ligand was 1:1, and in the case of NTA, it was 1:2. Equations (1) and (2) shown the complexation reaction

HfCl_4_ + Y^x−^ → [HfY]^4+x^ + 4Cl^−^, Y = DTPA, CDTA, dpta, HEDTA (1)

HfCl_4_ + 2Y^x−^ → [HfY]^4+x^ + 4Cl^−^, Y = NTA (2)

It is known that the hydrolysis of hafnium cations begins already at pH > 0, depending on the concentration of the metal in the solution [24,25]. This greatly complicates their complexation reactions, since for the best chelating effect the acids must be in a completely deprotonated form, which is achieved in a neutral medium. Monomeric hydrated Hf^4+^ occurs in dilute (≤10^−4^ mol dm^−3^) and strongly acidic (1 mol dm^−3^) aqueous solutions [26]. At higher concentrations of Hf(IV) hydroxocomplexes polymerize to form structures [Hf_4_(OH)_8_(H_2_O)_16_](OH)_8_ [17,27], shortly named HfO^2+^. Moreover, these species are formed during the hydrolysis appears and exist in the equilibrium with the solid phase. In [28] the authors supposed that there is an equilibrium between oxide forms as heterogenic fase and monomeric hafnium species in acid medium, which can appear due to hydroxide precipitation (Equation (3)):

(3) $${\mathrm{Hf}(\mathrm{OH})}_{2}^{2+}+2H_{2}O⇄{\mathrm{Hf}(\mathrm{OH})}_{4}+2H^{+}$$

The [Hf_4_(OH)_8_(H_2_O)_16_]^8+^ cation is probably destroyed during complexation with polyaminocarboxylic acids, but if [Hf_4_(OH)_8_(H_2_O)_16_](OH)_8_ structures are formed they do not enter into a complexation reaction which hinders getting the complexes.

Hafnium halides (chloride, bromide) and hafnium oxide-dichloride HfOCl_2_ were taken as the starting hafnium-containing substances. It does not matter whether HfCl_4_ or HfOCl_2_ is used. Using both of these salts leads to complex formation. However in the case of obtaining HfCDTA: when carrying out the synthesis with HfOCl_2_ at pH = 3–4, minimal precipitation is observed and the complex crystallizes from solution, while at pH = 6–7, a large amount of precipitate [Hf_4_(OH)_8_(H_2_O)_16_](OH)_8_ is observed. Moreover, the complex precipitates in the form of a gel with a high content of polymerized hafnium hydroxides. At the same time, when using hafnium bromide at pH = 6–7, excessive formation of hafnium hydroxides is not observed, and the product is isolated in high yield.

The synthesis was carried out by dissolving the required acid in water, bringing the pH to the required value by adding sodium hydroxide or sodium carbonate. In this case, one can use any of the proposed salts. To obtain HfHEDTA, the trisodium salt of the acid was dissolved in water and then the pH value was adjusted to the desired value by adding hydrochloric acid. After establishing the required value of the acidity of the medium, the hafnium salt was added. In some cases, even at a low pH, turbidity of the solution was still observed due to the precipitation of hydroxides.

The acids DTPA, NTA, DCTA, CDTA are poorly soluble in water, however, the addition of a hafnium salt without alkali at room temperature leads to the dissolution of the precipitate, which indicates the formation of complexes. However, large amounts of acid residues can be observed in the corresponding NMR spectra. Thus, under these conditions, the complexation reactions occurs partially with low yield, or require a large amount of time for completion.

#### 2.1.1. Na[HfDTPA]·3H_2_O (**1**)

The complex Na[HfDTPA]·3H_2_O can be obtained and isolated in high yield at pH = 3–4. In this case, no absorption bands are observed in the IR and NMR spectra either from the unreacted acid or from its sodium salt. At the same time, at pH = 6–7 complexation occurs partially due to the formation of an impurity (Na_5_DTPA), while under these conditions it is impossible to obtain a complex by crystallization from solution, since upon evaporation the mixture solidifies into a glassy mass. It should be noted that in this case the replacement of hafnium oxide-dichloride with hafnium chloride at pH = 6–7 did not lead to any improvement of the result.

#### 2.1.2. [HfCDTA(H_2_O)_2_]

When obtaining the complex [HfCDTA(H_2_O)_2_] by the interaction of HfOCl_2_ with the sodium salt of the acid, it is also necessary to carry out the synthesis in the range of pH = 3–5. An increase in pH to 7 leads to the formation of a large amount of hafnium hydroxides, which polymerizes at high metal concentrations and forms a gel-like mass, poorly soluble in water. However, the replacement of hafnium oxide-dichloride with chloride or bromide under these conditions leads to the formation of a pure complex without impurities, although in some cases turbidity of the solution may be observed, which is removed by filtration or centrifugation. This complex dissolves in 95% ethyl alcohol when boiled.

#### 2.1.3. Na_2_[Hf(NTA)_2_]·3H_2_O, Na_2_[Hf_2_(dpta)_2_], Hf-HEDTA

Unlike the previous complexes, the complexes Na_2_[Hf(NTA)_2_]·3H_2_O, Na_2_[Hf_2_(dpta)_2_] and Hf-HEDTA are obtained in a wider pH range 3–7 using any of the proposed hafnium-containing reagents. Most likely, the complexation in this case proceeds much faster than the hydrolysis of hafnium cations. In the case of NTA, even the dissolution of the precipitate of hafnium hydroxides is observed followed by its transformation into a complex, although the formation constant of the complexonate is much lower than the formation constant of hafnium hydroxide. It can also be assumed that the hydroxyl group, which takes part in complexation in the case of Na[Hf_2_(dpta)_2_] and Hf-HEDTA, acts as an additional stabilization factor. The participation of the hydroxyl group in the binding is clearly seen from the results of X-ray diffraction analysis in the case of dpta and in the NMR spectra in the case of HEDTA, since in the latter, the proton spectrum of the complex lacks a signal corresponding to the proton of the hydroxyl group.

### 2.2. Isolation and Purification

Complexes **1**–**4** were isolated from the solution by crystallization from a water-alcohol or water-acetone mixture. In this case, the addition of alcohol or acetone is necessary especially for small scale reactions, since these complexes are extremely soluble in water and crystallize only from highly concentrated solutions.

Upon evaporation, the main impurity is sodium chloride or bromide, which has a very similar solubility to the complexes. However, this problem is solved by prolonged boiling of the mixture in alcohol and recrystallization from it. In this case, it is advantageous to use hafnium bromide, since sodium bromide dissolves in alcohol better than chloride.

Thus, the acidity of the medium in the range from 3 to 7 has practically no effect on the formation of hafnium complexes with acids NTA, dpta, and HEDTA, while the preparation of complexes with acids DTPA and CDTA must be carried out in a medium with pH = 3–4. The formation of the complex is affected by the structure of the acid and the presence of additional stabilizing factors. Thus, nitrilotriacetic acid, being the smallest of the available acids, probably has the highest lability in comparison with the others, which allows us to assume that the ligand is more rapidly attached to the metal atom than the hydroxide ion. At the same time, the mobility and solubility of DTPA in water is limited by the presence of intramolecular hydrogen bonds [29]. Moreover, in the case of CDTA, the rate of complexation is limited by the cyclohexane ring [29]. Therefore, it is necessary to acidify the solution, providing hafnium cations to have no time to react with hydroxide ions, or use hafnium chloride or bromide directly. The acids dpta and HEDTA have a hydroxyl group, which is involved in complexation, providing additional stability of the complexes due to the formation of six-membered rings.

### 2.3. Physicochemical Properties

#### 2.3.1. IR Spectroscopy

The main sign of the formation of a complex is an intense broadened absorption band corresponding to COO^−^stretching vibrations observed at ~1600 cm^−1^ in the spectrum (Table 1). The spectra of the complexes also show a hypsochromic shift of the absorption band of the C=O stretching vibrations of carbonyl groups. In the complexes Na_2_[Hf(NTA)_2_]·3H_2_O, Na[HfDTPA]·3H_2_O, [HfCDTA(H_2_O)_2_], and Na[Hf_2_(DPTA)_2_], a broadening or appearance of a broad absorption band of stretching vibrations of OH groups from attached water molecules is observed at 3500 cm^−1^. Often in the spectra of complexes, the absorption bands of the carbonyl and carboxyl groups have several local maxima corresponding to their amount in the acid. This may indicate that, because of complexation, these groups become nonequivalent to each other, which is also confirmed by the NMR spectra.

For example, the absorption band of stretching vibrations of the carbonyl group in complex Na_2_[Hf(NTA)_2_]·3H_2_O broadens and shifts from 1731 to 1626 cm^−1^ and has three shoulders at 1673, 1651, and 1626 cm^−1^, while as in the spectrum of Na_3_NTA, two narrow bands are observed at 1672 and 1628 cm^−1^. The absorption band of stretching vibrations of the ionized carboxyl group appears at 1267 cm^−1^, and in the spectra of salts, this band is shifted towards higher wavenumbers.

#### 2.3.2. NMR Spectroscopy

**Figure 2:**
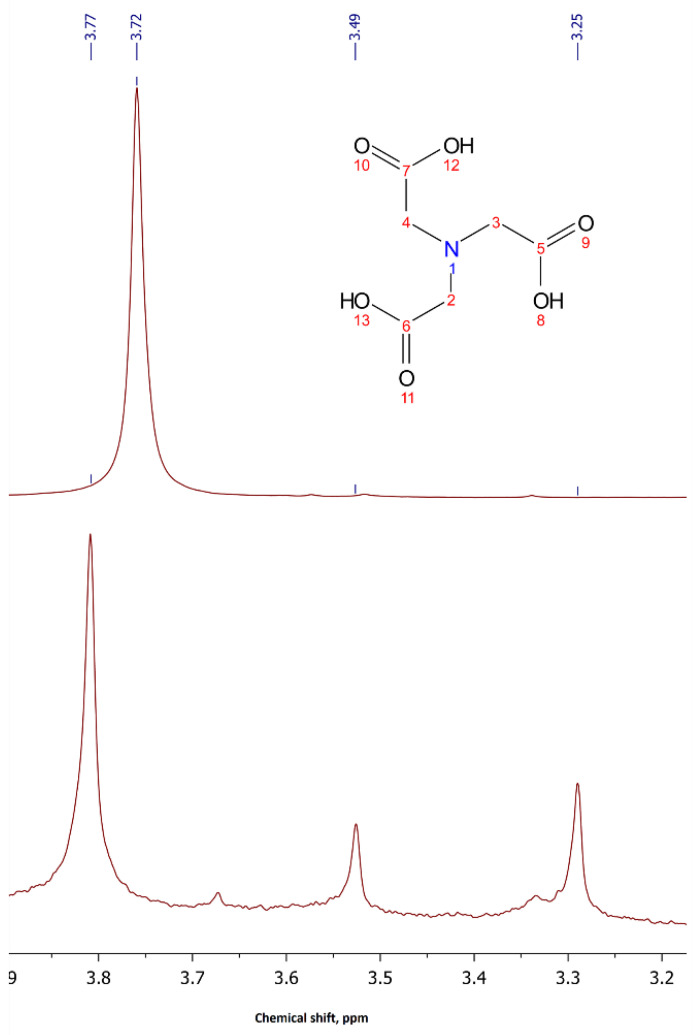
^1^H-NMR spectra of Na_3_NTA and Na_2_[Hf(NTA)_2_]·3H_2_O in D_2_O.

The formation of complexes was also confirmed by the results of ^1^H-NMR spectroscopy in D_2_O. The main indicator of complexation is the increase in the number of signals in the spectra compared to the spectrum of the acid or its salt. This is explained by the fact that complex formation leads to the formation of a rigid N-Hf and O-Hf bond. Therefore, the rotation of hydrogen atoms of methylene groups around the C-C bond is hampered, as a result of which degeneracy is completely removed from them [30,31]. Thus, in the spectrum of complex Na_2_[Hf(NTA)_2_]·3H_2_O, three signals are observed with an integral intensity ratio of 8:2:2 (Figure 2).

The situation is even more complicated in complexes with acids, which have a more branched structure. In the spectra of complexes there is a significant increase in the number of signals compared to the spectra of sodium salts of their acids. In this case, only deuterated water can be used as a solvent, since all complexes are insoluble in organic solvents. In this case, it is even more difficult to identify the belonging of the signals due to the intense hydrogen exchange.

**Figure 3:**
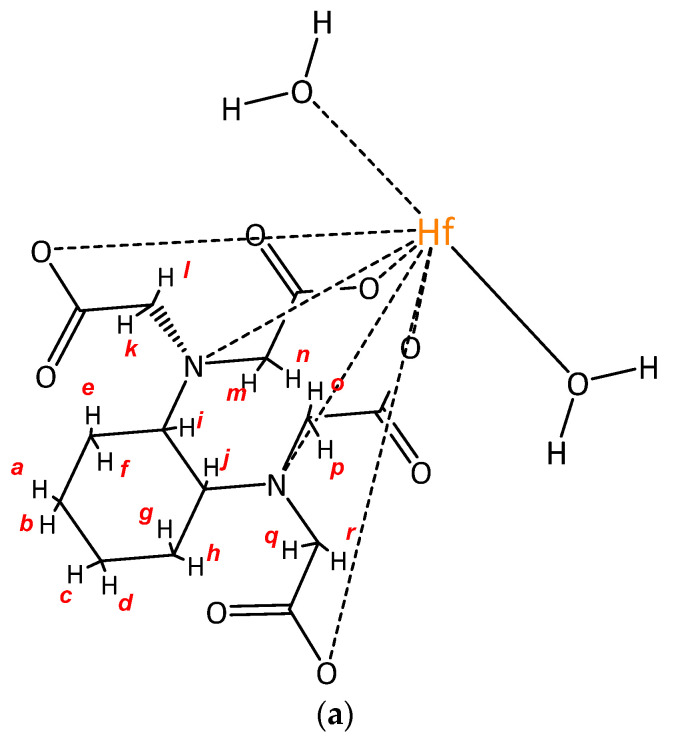

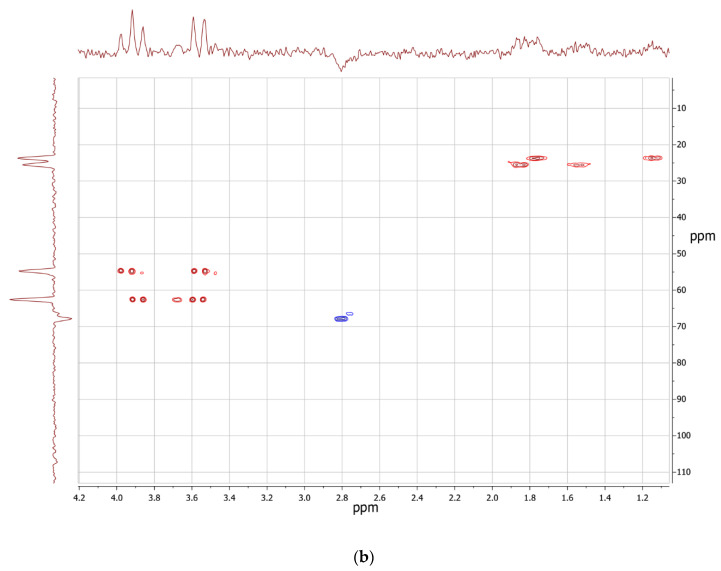
(**a**) Exemplary structure of the complex [HfCDTA(H_2_O)_2_]; (**b**) 2D ^1^H-^13^C HMBC NMR spectra of the complex [HfCDTA(H_2_O)_2_] in D_2_O solution.

In order to relate the signals the 2D ^1^H-^13^C HMBC NMR spectra have been reported. In (Figure 3) one can see the ^1^H- and ^13^C-NMR spectra 2D ^1^H-^13^C HMBC NMR spectra of [HfCDTA(H_2_O)_2_] (Figure 3b). The signals ^1^H/^13^C (1.15/23.67), (1.52/25.50), (1.75/1.87), (1.87/26.27) corresponds to *a-h* protons of CH_2_-groups of cyclohexane ring (Figure 3a), which are not equivalent in complex. Probably the conformation of cyclohexane ring also influences it. The ^1^H/^13^C (2.80/68.13) signal corresponds to *i-j* protons of CH-groups in cyclohexane ring. There is one signal so we can say that these two protons are equivalent. Two signals in the ^13^C-NMR spectrum at 179.20, 177.38 ppm indicate that two of the carboxylic groups in the complex are equivalent to the two other and the two signals of CH_2_-groups at 62.51, 67.79 ppm also confirm that. However in the ^1^H-NMR spectrum there is a group of signals at 3.91–9.53 ppm. It is known that in such complexes hydrogen atoms can be nonequivalent due to intensive hydrogen exchange or their hampered rotation around C-C bonds, but in the 2D ^1^H-^13^C HMBC NMR spectra there are four groups of signals ^1^H/^13^C (3.97, 3.91/54.74), (3.58, 3.52/54.74), (3.91, 3.86/62.57), (3.67, 3.59, 62.57). We suppose that there are eight doublets, which relate to the *k-r* protons in CH_2_-groups. Thus, the hydrogen atoms are non-equivalent to each other.

**Figure 4:**
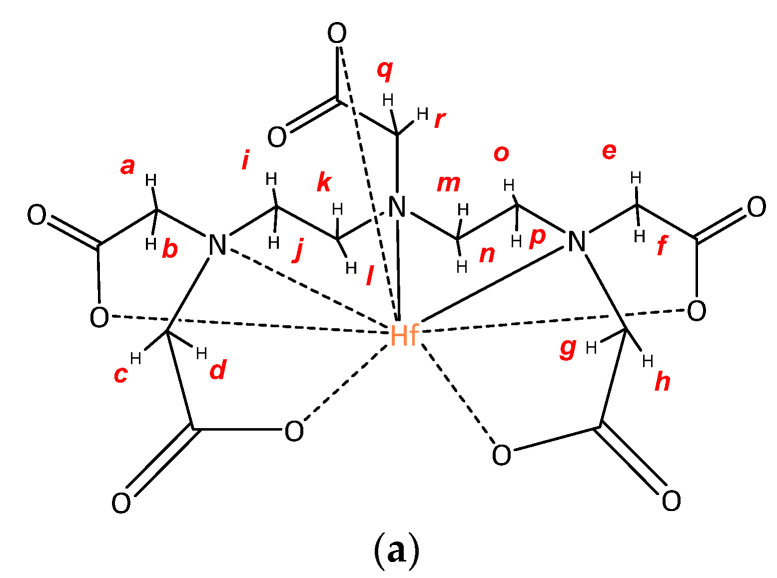

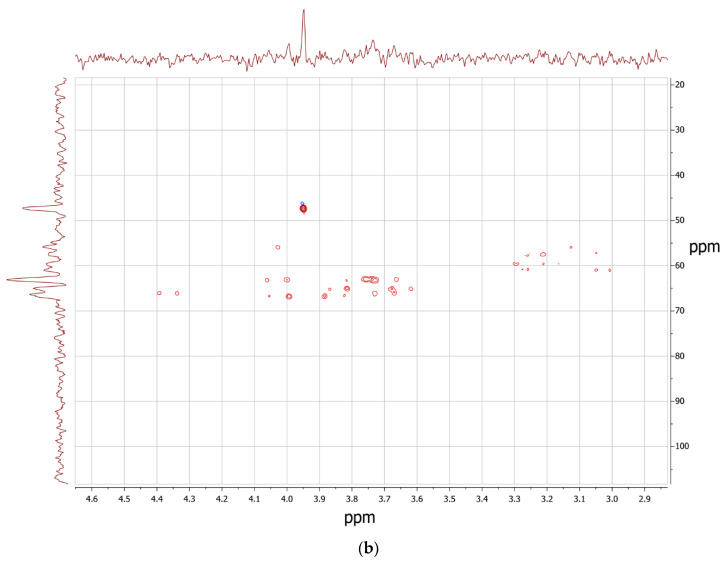
(**a**) Exemplary structure of the complex Na[HfDTPA]·3H_2_O; (**b**) 2D ^1^H-^13^C HMBC NMR spectra of Na[HfDTPA]·3H_2_O solution in D_2_O.

In the ^13^C-NMR spectrum of Na[HfDTPA]·3H_2_O (Figure S4) one can see five signals at 180.33, 180.15, 179.98, 179.10 and 168.99 which correspond to the C-atoms in the COO^−^ groups. The signals at ^1^H/^13^C (4.39/66.07) and (4.33/66.07) are doublets that presumably correspond to protons *q-r* at separate CH_2_-groups. The groups of signals at ^1^H/^13^C (4.05/63.15), (4.00/63.15), (3.99/66.76), (3.87/66.76), (3.80/65.00), (3.75/63.02), (3.61/65.00) correspond to the eight nonequivalent protons *a-d* of four CH_2_-groups (Figure 4a,b). In addition, a group of broad signals at (3.20/57.41) corresponds to the last eight protons *i-p* of methylene groups, which are nonequivalent due to their hampered rotation around the C-C bond. Unfortunately, due to intensive hydrogen exchange, probably with the solvent molecules, the proton signals are superimposed obstructing the decryption of the spectra.

**Figure 5:**
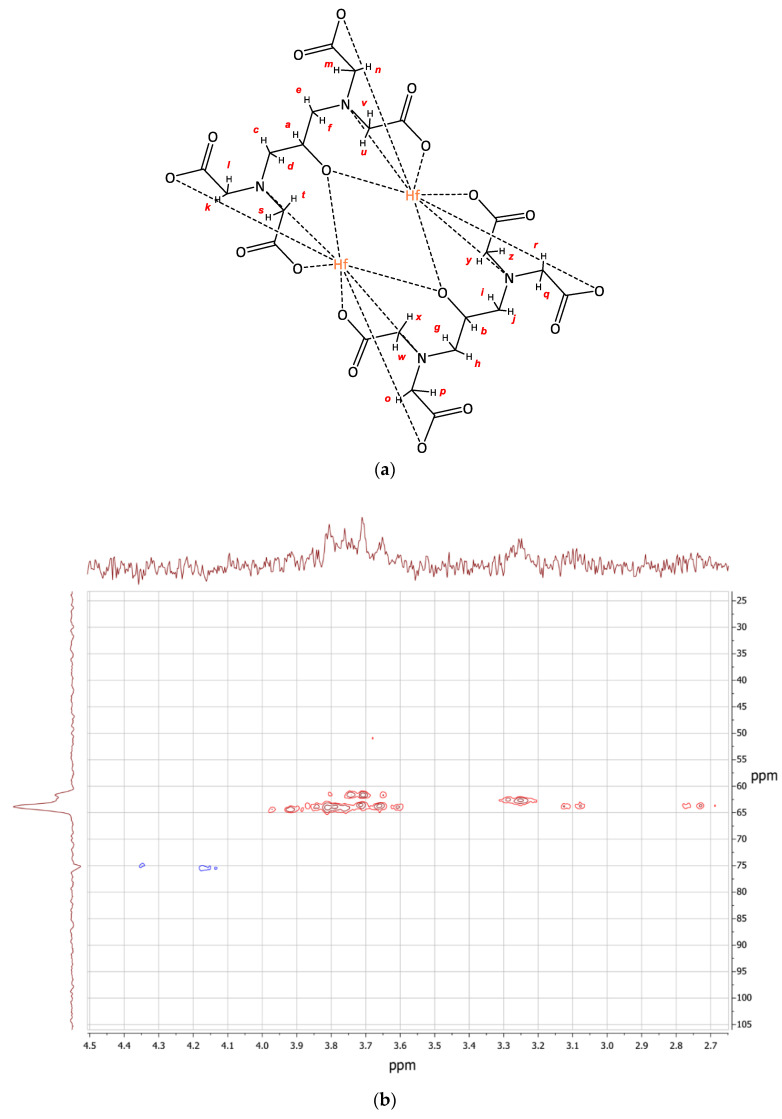
(**a**) Exemplary structure of the complex Na_2_[Hf_2_(dpta)_2_] 7.5H_2_O·0.5EtOH; (**b**) 2D ^1^H-^13^C HMBC NMR spectra of Table 2. Hf_2_(dpta)_2_] 7.5H_2_O·0.5EtOH solution in D_2_O.

The ^13^C NMR spectra of Na_2_[Hf_2_(dpta)_2_]7.5H_2_O·0.5EtOH (Figure S8) shows four signals at 180.82, 180.37, 179.39, 177.58 ppm corresponding to the C-atoms of its carboxylic acid groups. The four signals indicate that both of dpta ligands in the complex are equivalent to each other. However, there are two signals at 75.46 ppm, 74.95 ppm corresponding to the C-atoms of CH-O group. In the 2D ^1^H-^13^C HMBC NMR spectra there is a coincidence with the two triplets in the ^1^H-NMR spectra at 4.3, 4.16 ppm of protons *a-b* (Figure 5a). The signals at ^1^H/^13^C (3.28/62.5), (3.24/62.5), (3.11/63.7), (2.72/63.6) (Figure 5b) correspond to the protons *c-j* of the four CH_2_-groups according to [13]. In our ^1^H-NMR spectra, we have a group of signals at 3.96–3.60 ppm. According to [13] there should be four doublets corresponding to the CH_2_-group protons *k-r* which are nonequivalent. In addition, the most intensive ^1^H/^13^C signal (3.70/50.91) corresponds to the four equivalent protons *s-z* of the last CH_2_-groups.

**Figure 6:**
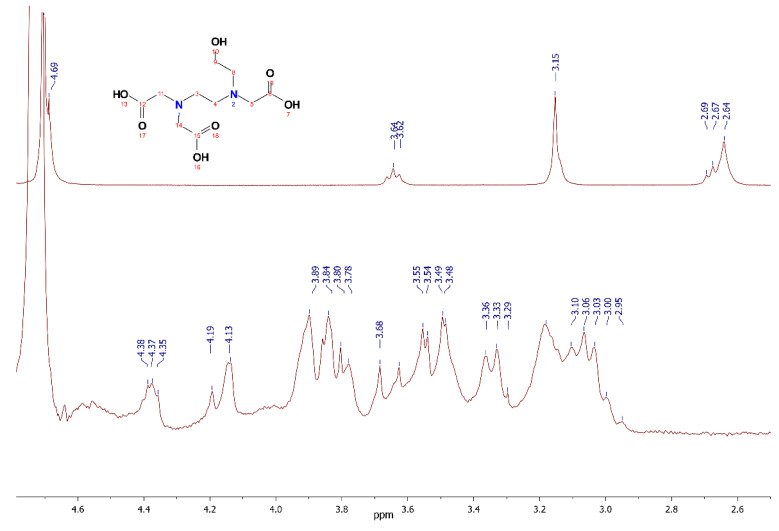
^1^H-NMR spectra of Na_3_HEDTA and Hf-HEDTA in D_2_O.

In the spectra of the Hf-HEDTA complex solution (Figure 6), a distinctive feature is the absence of a signal at δ = 4.37, corresponding to the hydroxyl group, which indicates its participation in binding. Unfortunately, the structure of this complex has not been established. In in ^1^H-NMR spectrum one can see a group of broad superimposed signals which make it difficult to establish any correspondence with the structural elements.

Thus, with an increase in the “branching” of the acid structure and an increase in the number of donor atoms in the complexes, the bond becomes stronger and the hydrogen exchange in aqueous solution increases significantly, resulting to an increased number of signals in the final spectrum. Moreover, the H_2_O molecules of the solvent can change the coordination sphere of the complexes and generate new hydrated species what result in an increase of the number the signals in the spectra. In this case, the chemical shift depends on pH of the medium: with an increase in pH, the signals shift to the strong field region.

#### 2.3.3. X-ray Diffraction

**Figure 7:**
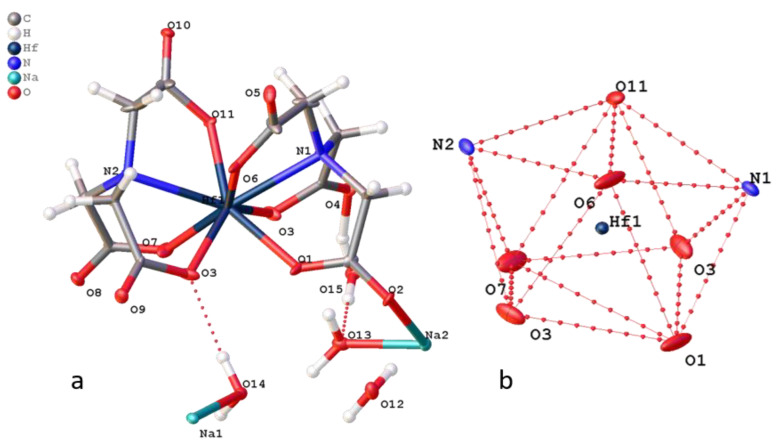
Structure of (**a**) **1** and (**b**) projection of the [HfN_2_O_6_] coordination polyhedron.

The structure of compound **1** contains the complex anion bis(nitrilotriacetato)hafnate(IV) [Hf(NTA)_2_]^2−^ (Figure 7), sodium cations, and four water molecules. Compound **1** crystallizes in the monoclinic system (space group C2/m), providing the mirror plane passed through the Hf1, O1, and O7 atoms, reflecting all other atoms in this plane. In addition, unlike the structure of (NH_4_)_2_[Hf(NTA)_2_] described by Held at el [32], the unit cell contains two nonequivalent NTA molecules with three common oxygen atoms. The anions have a similar structure; the N–Hf–N angle in the sodium salt is 129.6° versus 132.6° in the ammonium salt.

The hafnium atom is surrounded by two nitrogen atoms and six oxygen atoms (Figure 7b) from two different NTA molecules. The coordination polyhedron of the Hf1 atom is a distorted dodecahedron, in which the hafnium atom has the sp^3^d^4^ configuration [33].

**Figure 8:**
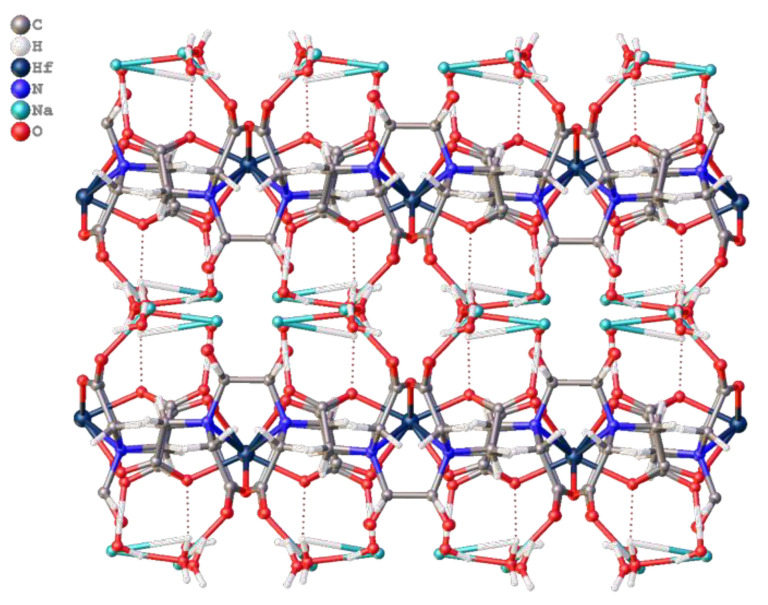
Cationic-anionic layers in crystal packing of **1**.

In the crystal packing of compound **1**, cationic-anionic layers are formed, built of anions [Hf(NTA)_2_]^2−^ and cations Na, and hydrated water molecules that are surrounded by sodium. The layers are connected due to strong hydrogen bonds of water molecules with the carboxyl groups of the anion and the oxygen atoms of NTA molecules included in the environment of sodium (Figure 8).

**Figure 9:**
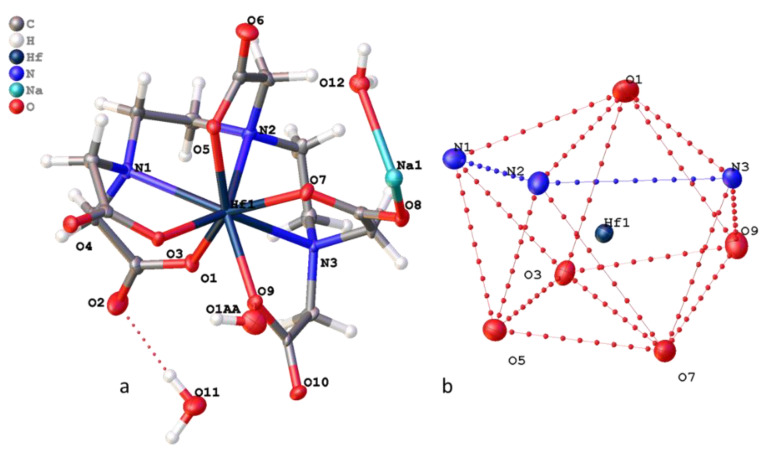
Structure of (**a**) **2** and (**b**) projection of the [HfN_3_O_5_] coordination polyhedron.

Compound **2** crystallizes in the monoclinic system (space group P21/c). The crystallographically independent part of the unit cell contains the [Hf(DTPA)]^−^ complex anion, sodium cation, and three water molecules (Figure 9). The hafnium atom is surrounded by three nitrogen atoms and six oxygen atoms of DTPA (Figure 1). The coordination polyhedron of the Hf1 atom is a distorted dodecahedron. Like the previously described complexes K[Hf(*DTPA*)] × 3H_2_O and K[Zr(*DTPA*)] × 3H_2_O, the position of the diethylenetriamine chain in DTPA complexes can be described as facial [20]. The dihedral angle between the planes N_1_HfN_2_ and N_2_HfN_3_ is 37º.

**Figure 10:**
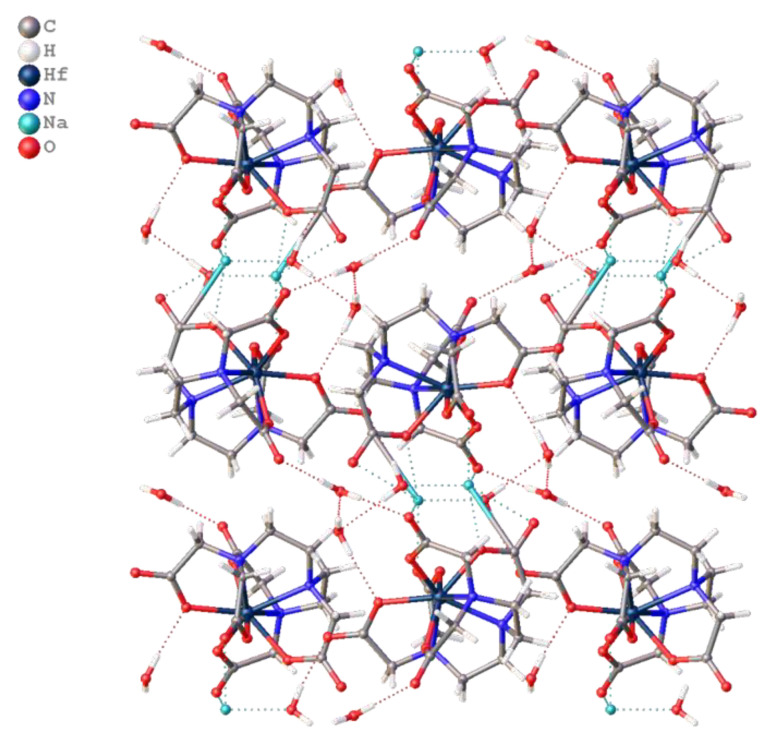
Cationic-anionic layers in the packing of **2**.

**Figure 11:**
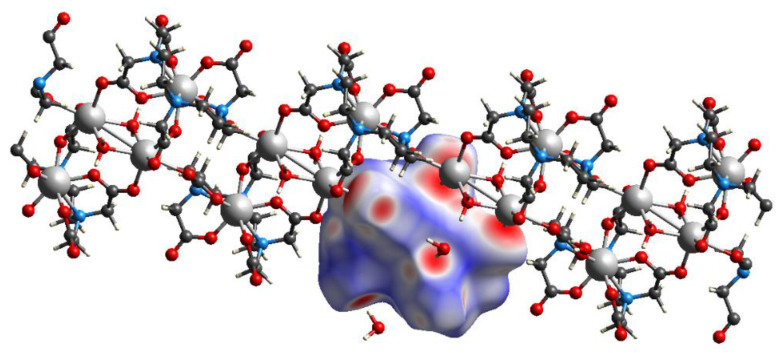
1D column in structure of **2** with visualization of the Hirshfeld surface for one [Hf(DTPA)]^−^ anion.

Like compound **1**, in the crystal packing of compound **2**, cationic-anionic layers are formed, built of [Hf(DTPA)]^−^ anions and Na^+^ cations, and hydrated water molecules that surround the sodium (Figure 10). However, in the structure of **2**, 1D polymer columns are formed during packing, the monomer unit of which can be taken as two Na^+^ ions located at a distance of 3.6 Å from each other, two hydrated water molecules, and four [Hf(DTPA)]^−^ anions linked by ionic bonds through hydroxyl groups with cations (Figure 11). The rest of the water molecules bond the columns in the package by hydrogen bonds.

**Figure 12:**
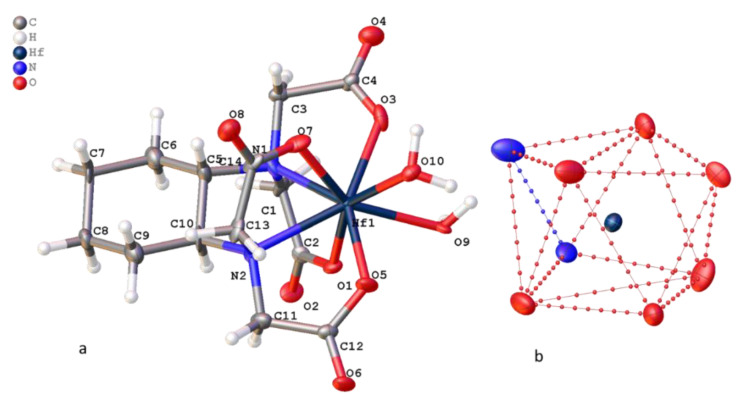
(**a**) Structure of **3** and (**b**) projection of coordination polyhedron [HfN_2_O_6_].

Compound **3** crystallizes in the orthorhombic system (space group Pnma); the mirror plane passes through the Hf atom and “folds” the molecule in half. The crystallographically independent part of the unit cell of the compound contains the [Hf(DCTA)(H_2_O)_2_] complex with a population of 0.5 for all atoms. The environment of the hafnium atom includes two nitrogen atoms and four oxygen atoms (Figure 12) of the DCTA ligand and two oxygen atoms from two water molecules. The coordination polyhedron of the Hf1 atom is a distorted quadrangular antiprism.

**Figure 13:**
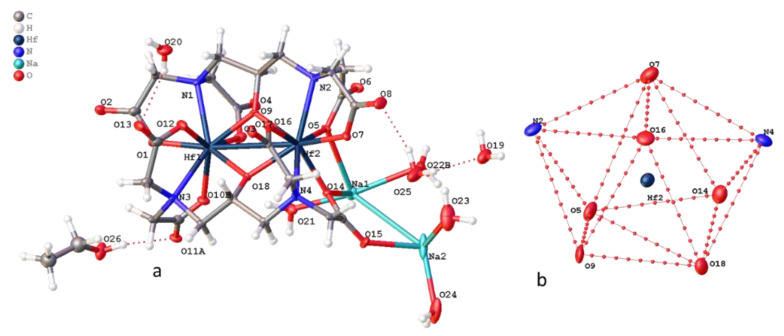
(**a**) Structure of **4** (**a**,**b**) projection of coordination polyhedron [HfN_2_O_6_] (**b**).

The structure of complex **4** indicates Na_2_[Hf_2_(dpta)_2_] 7.5H_2_O**^.^**0.5EtOH is isostructural to the previously described zirconium complex Na_2_[Zr_2_(dpta)_2_]·7H_2_O**^.^**C_2_H_5_OH [13]. The structure of the [Hf_2_(dpta)_2_]^2−^ anion in complex **4** is identical to [Zr_2_(dpta)_2_]^2−^: each of the two dpta ligands in the [Hf_2_(dpta)_2_]^2−^ anion binds to two Hf atoms by bridging oxygen atoms from an alkoxide group, the oxygen atoms of carboxyl groups and nitrogen atoms N atoms, giving an 8-coordinated Hf atom (Figure 13). The environment of the hafnium atoms in the complex is a distorted horizontal two-cap trigonal prism. The torsion angle O9–Hf1–Hf2–O18 is greater than the corresponding angle in the zirconium complex by 6° and is 18.5°. Two hafnium centers are almost equivalent with non-crystallographic mirror symmetry through two alkoxide oxygen atoms and carbon atoms of methine groups (torsion angle C6-O9-O18-C17 1.8°). The two ligands have different conformations: one is more expanded than the second ligand. The O(O9) alkylide atom from the first ligand lies in the plane built of two nitrogen atoms (N1 and N2) and two hafnium atoms (RSMD N1Hf1Hf2N2 plane = 0.053 Å, O9-the plane distance = 0.013 Å). In the second ligand, two nitrogen atoms (N3 and N4) and two hafnium atoms also lie in the plane (RSMD N3Hf1Hf2N4 plane = 0.026 Å), but the alkoxide plane is O18 distance = 0.316 Å. This plane intersects the plane of the first ligand at an angle of 147°. A similar angle in the zirconium complex is 136°.

**Figure 14:**
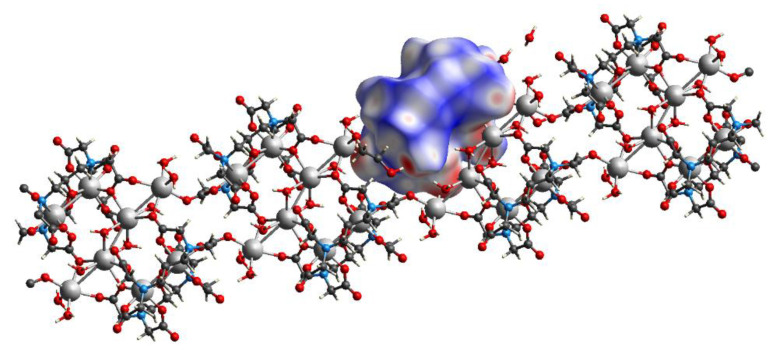
1D column in structure of **4** with visualization of Hirshfeld surface for one [Hf_2_(dpta)_2_]^2−^ anion.

When the crystals are packed, two [Hf_2_(dpta)_2_]^2−^ anions are linked into pairs due to coordination of carboxylate oxygen atoms with two bridging sodium cations, which in turn form polymer columns due to coordination with two more sodium cations (Figure 14). The latter are linked together by hydrogen bonds with water molecules and CH···O contacts.

The lengths of the Hf–O bonds in all compounds have similar values (Table 2), including water molecules in compound **3** and alkoxy oxygen atoms in compound **4**. The average value of the Hf–O bond is 2.143 Å for compound **1**, 2.124 Å for compound **2**, 2.151 Å for carboxyl atoms and 2.180 Å for water molecules in compound **3** and 2.130 Å for carboxyl and 2.160 Å for alkoxy oxygen atoms in **4**. Bond lengths Hf–N is longer on average by 0.3 Å and amounts to 2.410, 2.416, 2.315, and 2.442 Å for compounds **1**–**4**, respectively. The length of the Hf–Hf contact in compound **4** is 3.5694(3), which is slightly longer than those published in the [34] values ranging from 3.089 to 3.498 Å.

## 3. Biological Activity

The complex [HfCDTA(H_2_O)_2_] (**3**) was subjected to further biological testing. Intravenous injection of a 0.17 M solution of a hafnium complex in saline showed that the half-lethal dose for this solution is 281 ± 55 mg Hf/kg, and the maximum tolerated dose of MTD = 118 ± 33 mg Hf/kg. A study of the acute toxicity of the complex in 5% glucose solution showed that for intravenous injection, the half-lethal dose is LD 50 = 362 ± 59 mg Hf/kg, the maximum tolerated dose of MTD = 185 ± 35 mg Hf/kg, which is significantly higher than for the complex dissolved in saline. At the same time, the drug showed good contrasting ability when administered intratumorally.

## 4. Experimental Section

### 4.1. General Information

Starting reagents (HfCl_4_, HfOCl_2_, DTPA, CDTA, NTA, dpta, and HEDTA) and solvents with a basic substance content of at least 97% were obtained from Sigma-Aldrich (Moscow, Russia) and used without additional purification.

Instrumental analysis methods: ^1^H-NMR spectra of the solutions of the investigated compounds were recorded on an Avance II-300 spectrometer (Bruker, Moscow, Russia) at a frequency of 300.21 MHz with internal deuterium stabilization; tetramethylsilane was used as external standard.

IR spectra of the synthesized compounds were recorded on an INFRALUM FT-02 IR Fourier spectrometer (NPF AP Lumex, Moscow, Russia) in the range 4000–400 cm^−1^ with a resolution of 1 cm^−1^; samples were prepared as KBr pellets.

X-ray diffraction analysis of compounds **1**–**4** was performed on a Bruker APEX-II CCD instrument, λ_Mo_ = 0.71073 Å. The data have been corrected for absorption based on the measurements of the equivalent reflections. The structures were solved by direct methods and refined by the method of least squares of the full matrix in F^2^ with anisotropic thermal parameters for all non-hydrogen atoms using the SHELX program [35] included in the OLEX2 program package [36]. All hydrogen atoms are placed in calculated positions and refined using the rider model.

Crystallographic data, details of data collection, and results of structure refinement are summarized in Table S1 Crystallographic data for compounds **1**–**4** have been deposited with the Cambridge Crystallographic Data Center as supplementary publications (CCDC nos. 2046772-2046775).

To analyze the Hirschfeld surface, we used the Crystal Explorer 17.5 program [37]. Donor-acceptor groups are rendered using standard (high) surface resolution, and d_norm_ surfaces are displayed on a fixed color scale from −0.640 (red) to 0.986 (blue) au.

### 4.2. Synthesis

#### 4.2.1. Na_2_[Hf(NTA)_2_]·3H_2_O (**1**)

A weighed portion of NTA (2 g, 10.46 mmol) with a small amount of sodium hydroxide (1.25 g; 31.38 mmol) was dissolved in 200 mL of water, after which the pH of the solution was adjusted to 5.5 by adding 0.3 M sodium hydroxide or hydrochloric acid solutions as needed. After establishing the required pH value, a weighed portion of HfOCl_2_·8H_2_O (2.14 g; 5.23 mmol) was added, with a slight turbidity being noted. The solution was boiled for 3 h and evaporated to 30 mL. After cooling to room temperature, the solution was centrifuged at 3600 rpm for 10 min (or filtered through a membrane filter) and decanted. The resulting solution was evaporated to 10 mL and precipitated with ethyl alcohol. The crystals obtained were washed with 95% ethyl alcohol, filtered off and boiled in alcohol for 30 min. The suspension was cooled; the precipitate was filtered off and dried. As a result, 2.82 g of a white crystalline powder was obtained. Yield, 70%. IR (KBr): ν_OH_: 3600, 3496, 3182 cm^−1^, ν_CH_ 2975, 2924, 2903 cm^−1^, ν_OH(H-bond)_ 2632, 2319, 2585 cm^−1^, ν_C=O_ 1651, 1673, 1626 cm^−1^, ν_COO_ 1267, 1340 cm^−1^. ^1^H-NMR (D_2_O) ^1^H δ 3.67; 3.53; 3.29; 1.98 (s, H1-12). Anal. C 20.95%; H 2.97%, N 4.6% Hf 26.4%. The crystals were studied by X-ray diffraction.

#### 4.2.2. Na[HfDTPA]·3H_2_O (**2**)

A weighed portion (2 g; 5.08 mmol) of DTPA with a small amount of sodium hydroxide (0.6 g; 15.24 mmol) was dissolved in 200 mL of water, after which pH of the solution was adjusted to 3.5 by adding 0.3 M sodium hydroxide or hydrochloric acid solutions. After establishing the required pH value, a weighed portion of HfOCl_2_·8H_2_O (2.1 g; 5.08 mmol) was added, while a slight turbidity was noted. The solution was boiled for 3 h and evaporated to 30 mL. After cooling to room temperature, the solution was centrifuged at 3600 rpm for 10 min (or filtered through a membrane filter) and decanted. The resulting solution was evaporated to 10 mL and precipitated with acetone. The crystals obtained were washed with 95% ethyl alcohol, filtered off and boiled in alcohol for 30 min. The suspension was cooled, the precipitate was filtered off and dried. As a result, 1.8 g of a white crystalline powder was obtained. Yield, 45%. IR (KBr) ν_OH_ 3471, 3358, ν_CH_ 3006, 2986, 2960, 2922, 2878, ν_OH(H-bond)_ 2683, 2640, 2149, ν_C=O_ 1665, ν_COO_ 1475, 1464, 1436, 1349, 1326. ^1^H-NMR (D_2_O) ^1^H δ 4.39; 4.33; 4.05; 3.99; 3.88–3.62 (m, *J*_H-H_ = Hz), 3.48–2.98 (m, J_H-H_ = Hz). Anal. C 26.26%, H 4.07%, N 6.6%; Hf 24.4%. The crystals were studied by X-ray diffraction.

#### 4.2.3. [HfCDTA(H_2_O)_2_] (**3**)

Procedure 1. A weighed portion (2 g; 5.49 mmol) of CDTA with a small amount of sodium hydroxide (0.4 g; 10.98 mmol) was dissolved in 200 mL of water, after which the pH value of the solution was adjusted to 3.5 by addition of 0.3 M sodium hydroxide or hydrochloric acid solutions. After establishing the required pH value, a weighed portion of HfOCl_2_·8H_2_O (2.2 g; 5.49 mmol) was added, while a slight turbidity was noted. The solution was boiled for 3 h and evaporated to 30 mL. After cooling to room temperature, the solution was centrifuged at 3600 rpm for 10 min (or filtered through a membrane filter) and decanted. The resulting solution was evaporated to 10 mL and precipitated with acetone. The compound was recrystallized from 95% ethanol. The crystals were filtered off. As a result, 1.4 g of a white crystalline powder was obtained. Yield, 35%. IR (KBr) ν_OH_ 3429, 3234 cm^−1^, ν_CH_: 2983, 2934, 2864 cm^−1^, ν_OH(H-bond)_: 2405 cm^−1^, ν_C=O_ 1653 cm^−1^, ν_COO_ 1402, 1374, 1343, 1319 cm^−1^. NMR (D_2_O): ^1^H δ: 3.89 (t, *J* = 17 MHz), 3.56 (d, *J* = 18 MHz), 3.26 (s), 2.78 (t, *J* = 4 MHz), 1.84–1.72 (m, *J* = MHz), 1.52 (d), 1.19–1.04 (m). Anal. C 26.69%, H 4.25%, N 4.45%, Hf 24.8%. The crystals were studied by X-ray diffraction.

Procedure 2. Weighed portions (2 g; 5.49 mmol) of CDTA and HfBr_4_ (2.7 g; 5.49 mmol) were dissolved in 200 mL of water, after which the pH of the solution was adjusted to 7 by adding a 0.3 M sodium hydroxide solution. When turbid, the solution was filtered through a membrane filter, then boiled for 3 h and evaporated on a rotary evaporator. The powder was thoroughly washed with 90% ethyl alcohol. As a result, 2 g of a white crystalline powder was obtained. Yield, 42%. IR (KBr) ν_OH_ 3429, 3234 cm^−1^, ν_CH_: 2983, 2934, 2864 cm^−1^, ν_OH(H-bond)_: 2405 cm^−1^, ν_C=O_ 1653 cm^−1^, ν_COO_ 1402, 1374, 1343, 1319 cm^−1^. NMR (D_2_O): ^1^H δ: 3.89 (t, *J* = 17 MHz), 3.56 (d, *J* = 18 MHz), 3.26 (s), 2.78 (t, *J* = 4 MHz), 1.84–1.72 (m, *J* = MHz), 1.52 (d), 1.19–1.04 (m). Anal. C 26.69%, H 4.25%, N 4.45%, Hf 24.8%.

#### 4.2.4. Na_2_[Hf_2_(dpta)_2_] 7.5H_2_O 0.5EtOH (**4**)

A weighed portion (2 g; 6.21 mmol) of dpta with a small amount of sodium hydroxide (0.9 g; 24 mmol) was dissolved in 200 mL of water, after which the pH of the solution was adjusted to 5 by adding 0.3 M solutions of sodium hydroxide or hydrochloric acid. After establishing the required pH value, a weighed portion of HfOCl_2_·8H_2_O (2.5 g; 6.21 mmol) was added, while a slight turbidity was noted. The solution was boiled for 3 h and evaporated to 30 mL. The solution was cooled to room temperature, centrifuged at 3600 rpm for 10 min and decanted or filtered through a membrane filter. The resulting solution was evaporated to 10 mL and precipitated with by adding acetone. The crystals were filtered off. As a result, 1.2 g of a white crystalline powder was obtained. Yield, 30%. IR (KBr) ν_OH_ 3444 cm^−1^, ν_CH_ 2971, 2940, 2888 cm^−1^, ν_C=O_ 1670, 1648 cm^−1^, ν_COO_: 1381, ν_CN_ 1344 cm^−1^. ^11^H-NMR (D_2_O) δ: 4.32; 4.15; 3.94–3.50 (m, *J* = 4 MHz), 3.26–2.22 (m, *J* = 4 MHz), 3.09–3.05 (m, *J* = 4 MHz), 2.73–2.65 (m, *J* = 4 MHz). Anal. C 23.01%, H 3.47%, N 4.60%, Hf 29.95%. The crystals were studied by X-ray diffraction.

#### 4.2.5. HfHEDTA (**5**)

A weighed portion (2 g; 5.08 mmol) of sodium salt of HEDTA was dissolved in 200 mL of water, after which pH of the solution was adjusted to 5 by adding a 0.3 M hydrochloric acid solution. After establishing the required pH value, a weighed portion of HfOCl_2_·8H_2_O (2.1 g; 5.08 mmol) was added, while a slight turbidity was noted. The solution was boiled for 3 h and evaporated to 30 mL. The solution was cooled to room temperature, centrifuged at 3600 rpm for 10 min (or filtered through a membrane filter), and decanted. The resulting solution was evaporated on a rotary evaporator. As a result, a glassy mass was obtained, which solidified upon cooling. After grinding, the reaction mass was washed with isopropyl alcohol. The result was 1.8 g of a white crystalline powder being complex with a large amount of NaCl. Yield, 45%. IR (KBr) ν_OH_ 3454 cm^−1^, ν_CH_ 2972, 2934 cm^−1^, ν_C=O_ 1652, 1627, 1569 cm^−1^, ν_COO_ 1386 cm^−1^, νCN 1348 cm^−1^. ^11^H-NMR (D_2_O) δ: 4.37; 4.19; 4.14; 3.89–3.78 (m, *J* = 4 MHz), 3.68–3.48 (m, *J* = 4 MHz), 3.36–3.33 (m, *J* = 4 MHz); 3.18–3.00 (m, *J* = 4 MHz). Anal. C, 16.71%, H 6.11%, N 3.98%, Hf 17.3%. The crystals were studied by X-ray diffraction.

## 5. Conclusions

Thus, the hydrolysis of hafnium cations is not necessarily the determinant of the reaction conditions. The structure of the ligand plays a significant role. A slight “branching” reduces the probability of intramolecular hydrogen bonds, which hinder the ligand lability, as in the case of DTPA, and promotes the feasibility of the hydrolysis reaction, which is clearly seen on the example of complex Na_2_[Hf(NTA)_2_]·3H_2_O. On the other hand, this factor is not the only one determining the rate of the complexation reaction. The presence of closed cycles with a minimum of intramolecular bonds also limits the ligand lability, as in the case of CDTA. However, in this case, complexation proceeds much better than with DTPA. The presence of additional stabilizing factors significantly increases the probability of hydrolysis outstripping. In this case, such a factor is the hydroxyl group in the dpta and HEDTA ligands. These assumptions are fully consistent with the conditions under which complexes are obtained in high yields. Compounds Na_2_[Hf(NTA)_2_]·3H_2_O, Na[Hf_2_(DPTA)_2_], and Hf-HEDTA can be isolated in high yield in a wide range of acidity of the medium, while for complexes Na[HfDTPA]·3H_2_O and [HfCDTA(H_2_O)_2_] this range is significantly narrowed.

## Figures and Tables

**Table 1:** Selected absorption bands of stretching vibrations of complexes and corresponding acids.

Compound	ν(O-H), cm^−1^	ν(C=O) cm^−1^	ν(COO-), cm^−1^	Compound	ν(O-H), cm^−1^	ν(C=O) cm^−1^
Na_2_[Hf(NTA)_2_]·3H_2_O	3600, 3496, 3182,	1651, 1673, 1626 (vs)	1267, 1340 (vs)	NTA	3440	1731
Na[HfDTPA]·3H_2_O	3471, 3358 (vs)	1653 (vs)	1402, 1374, 1343, 1319 (vs)	DTPA	3084, 3020	1701, 1633
[HfCDTA(H_2_O)_2_]	3429, 3234	1653	1402, 1374, 1343, 1319	CDTA·H_2_O	3509, 3361, 3285	1661, 1709
Na_2_[Hf_2_(dpta)_2_]	3444	1670, 1648	1381	dpta	3441, 3155, 3031, 3011, 2975, 2731	1722
Hf-HEDTA	3454	1652, 1627, 1569	1386	HEDTA	3502	1731

**Table 2:** Selected bond lengths for compounds **1**–**4**, Å.

Bond	Compound				
	1	2	3	4	4
Hf–Hf	–	–	–	3.5694(3)	
Hf–N1	2.421(5)	2.459(3)	2.248(7)	2.435(4)	2.424(4)
Hf–N2	2.457(5)	2.373(3)	2.382(17)	2.457(4)	2.451(4)
Hf–N3	–	2.451(3)	–	–	–
Hf–O1	2.129(3)	2.097(3)	2.102(14)	2.128(3)	2.149(3)
Hf–O2	2.165(2)	2.105(3)	2.248(7)	2.114(3)	2.111(3)
Hf–O3	2.165(2)	2.137(3)	2.105(10)	2.125(3)	2.152(3)
Hf–O4	2.118(3)	2.119(3)	2.117(11)	2.16(2) 2.215(19)	2.134(3)
Hf–O5	2.137(5)	2.162(3)	2.243(14) *	2.155(3) **	2.131(3) **
Hf–O6	2.111(4)	–	2.195(15) *	2.138(3) **	2.174(3) **

* Hf–OH2, ** Hf–O (alkoxide).

## Data Availability

The data (spectroscopy data, X-ray data, ESI data) used to support the findings of this study are included within the article.

## Supplementary Materials

The following are available online, Figure S1: ^1^H NMR spectrum of crystal solution Na_2_[Hf(NTA)_2_]·3H_2_O (**1**) in D_2_O at 298 K, Figure S2. ^13^C NMR spectrum of crystal solution Na_2_[Hf(NTA)_2_]·3H_2_O (**1**) in D_2_O at 298 K, Figure S3. ^1^H NMR spectrum of crystal solution Na[HfDTPA]·3H_2_O (**2**) in D_2_O at 298 K, Figure S4. ^13^C NMR spectrum of crystal solution Na[HfDTPA]·3H_2_O (**1**) in D_2_O at 298 K, Figure S5. ^1^H NMR spectrum of crystal solution [HfCDTA(H_2_O)_2_] (**3**) in D_2_O at 298 K, Figure S6. ^13^C NMR spectrum of crystal solution [HfCDTA(H_2_O)_2_] (**1**) in D_2_O at 298 K, Figure S7. ^1^H NMR spectrum of crystal solution Na[Hf_2_(dpta)_2_]·7.5H_2_O·0.5C_2_H_5_OH (**4**) in D_2_O at 298 K, Figure S8. ^13^C NMR spectrum of crystal solution Na[Hf_2_(dpta)_2_]·7.5H_2_O·0.5C_2_H_5_OH (**1**) in D_2_O at 298 K, Figure S9. ^1^H NMR spectrum of solution HfCl4 and Na_3_HEDTA (**5**) in D_2_O at 298 K, Table S1: Crystallographic data and experimental and refinement details for compounds (**I**–**III**), Table S2. Crystallographic data and experimental and refinement details for compounds (**IV**).

## Sample Availability

Samples of the compounds are available from the authors.
