# Supplementary material for: Synthesis of Hafnium(IV) Polyaminoacetates
Source: Molecules. 2021 Jun 18;26(12):3725. doi: 10.3390/molecules26123725 (PMC8234123; doi:10.3390/molecules26123725)
Supplement: Supplementary file 1 [file molecules-26-03725-s001.zip › molecules-1244490-supplementary.pdf]

Supplementary data

Synthesis of Hafnium(IV) Polyaminoacetates

Alexandra T. Shulyak [1,2,*], Evgeniy O. Bortnikov [3], Alexey S. Kubasov [1], Nikita A. Selivanov [1], Alexey A. Lipengolts [4], Andrey P. Zhdanov [1], Alexander Yu. Bykov [1], Konstantin Yu. Zhizhin [1] and Nikolai T. Kuznetsov [1]

[1] Kurnakov Institute of General and Inorganic Chemistry, Russian Academy of Sciences, Leninskiy pr. 31, Moscow 119991, Russia; fobosax@mail.ru (A.S.K.); govee@yandex.ru (N.A.S.); zhdanov@igic.ras.ru (A.P.Z.); bykov@igic.ras.ru (A.Y.B.); zhizhin@igic.ras.ru (K.Y.Z.); ntkuz@igic.ras.ru (N.T.K.), at.shulyak@yandex.ru

[2] MIREA-Russian Technological University, Moscow State University of Fine Chemical Technologies, Lomonosov Institute of fine chemical technologie, pr. Vernadskogo, 86, Moscow 119454, Russia

[3] Weizmann Institute of Science, Organic Chemistry, 234 Herzl Street, Rehovot 7610001, Israel; bortnikoevol@gmail.com

[4] Federal State Budgetary Institution «N.N. Blokhin National Medical Research Center of Oncology» of the Ministry of Health of the Russian Federation (N.N. Blokhin NMRCO), Kashirskoye Shosse 24, Moscow 115478, Russia; lipengolz@gmail.com

* Correspondence: at.shulyak@yandex.ru; Tel.: +7-(916)-823-01-57

1. Experimental

Initial reagents and solvents. $HfCl_4$, $HfOCl_2$, DTPA, CDTA, NTA, dpta, and HEDTA (Aldrich) with a basic substance content of at least 97% were used without additional purification.

Instrumental analysis methods. ^1H NMR spectra of the solutions of the investigated compounds were recorded on a Bruker Avance II-300 spectrometer at a frequency of 300.21 MHz with internal deuterium stabilization; tetramethylsilane was used as external standards.

IR spectra of the synthesized compounds were recorded on an INFRALUM FT-02 IR Fourier spectrometer (NPF AP Lumex) in the range 4000–400 cm^{-1} with a resolution of 1 cm-1; samples were prepared in KBr pellets.

X-ray diffraction analysis of compounds **1-4** was performed on a Bruker APEX-II CCD instrument, λ_{Mo} = 0.71073 Å. The data have been corrected for absorption based on the measurements of the equivalent reflections. The structures were solved by direct methods and refined by the method of least squares of the full matrix in F^2 with anisotropic thermal parameters for all non-hydrogen atoms using the SHELX program included in the OLEX2 program package. All hydrogen atoms are placed in calculated positions and refined using the rider model.

Crystallographic data, details of data collection, and results of structure refinement are summarized in Table 3. Crystallographic data for **1–4** have been deposited with the Cambridge Crystallographic Data Center as supplementary publications (CCDC nos. 2046772-2046775).

To analyze the Hirschfeld surface, we used the Crystal Explorer 17.5 program. Donor-acceptor groups are rendered using standard (high) surface resolution, and d_{norm} surfaces are displayed on a fixed color scale from -0.640 (red) to 0.986 (blue) au.

2. NMR spectra

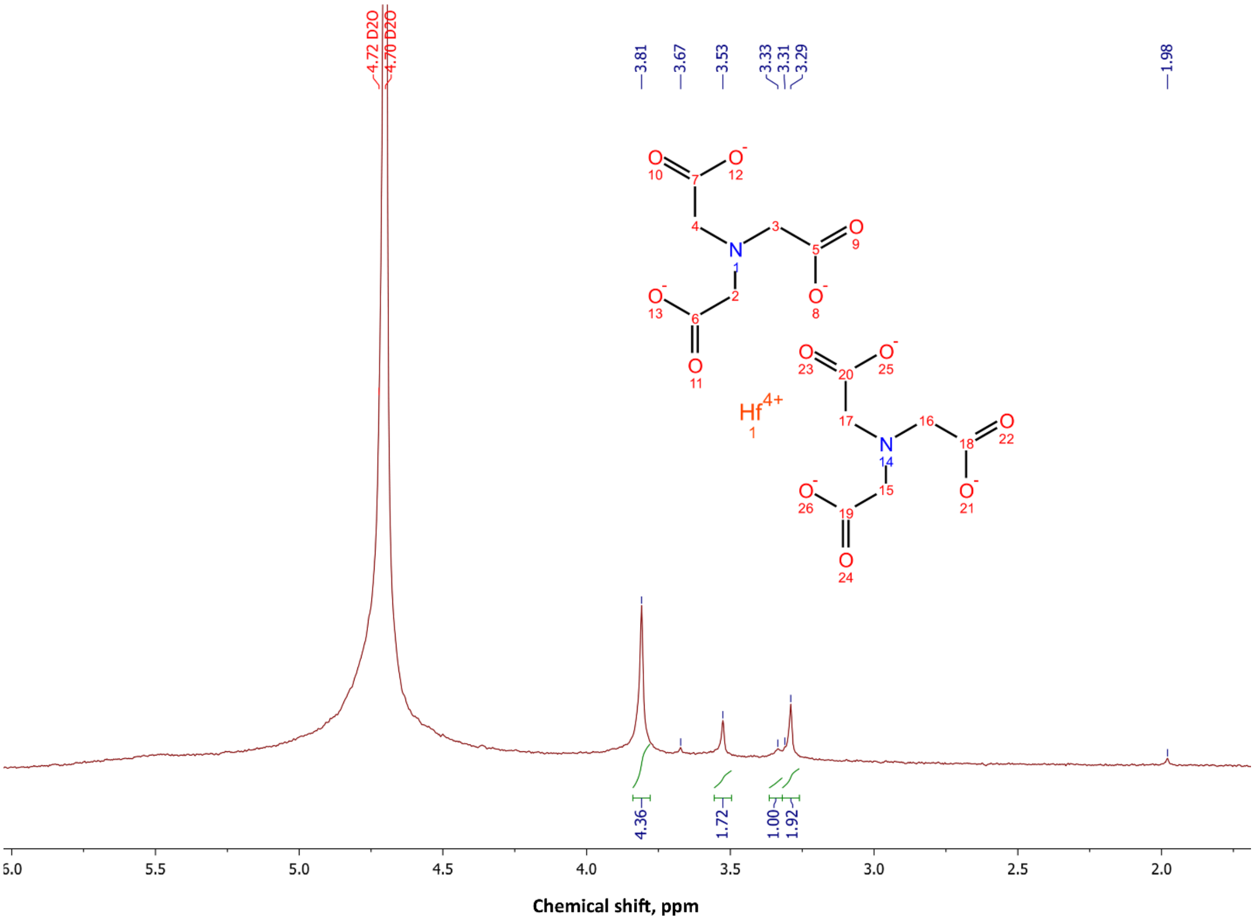

Figure S1. ^{1}H NMR spectrum of crystal solution $Na_2[Hf(NTA)_2]\cdot 3H_2O$ (1) in D_2O at 298K

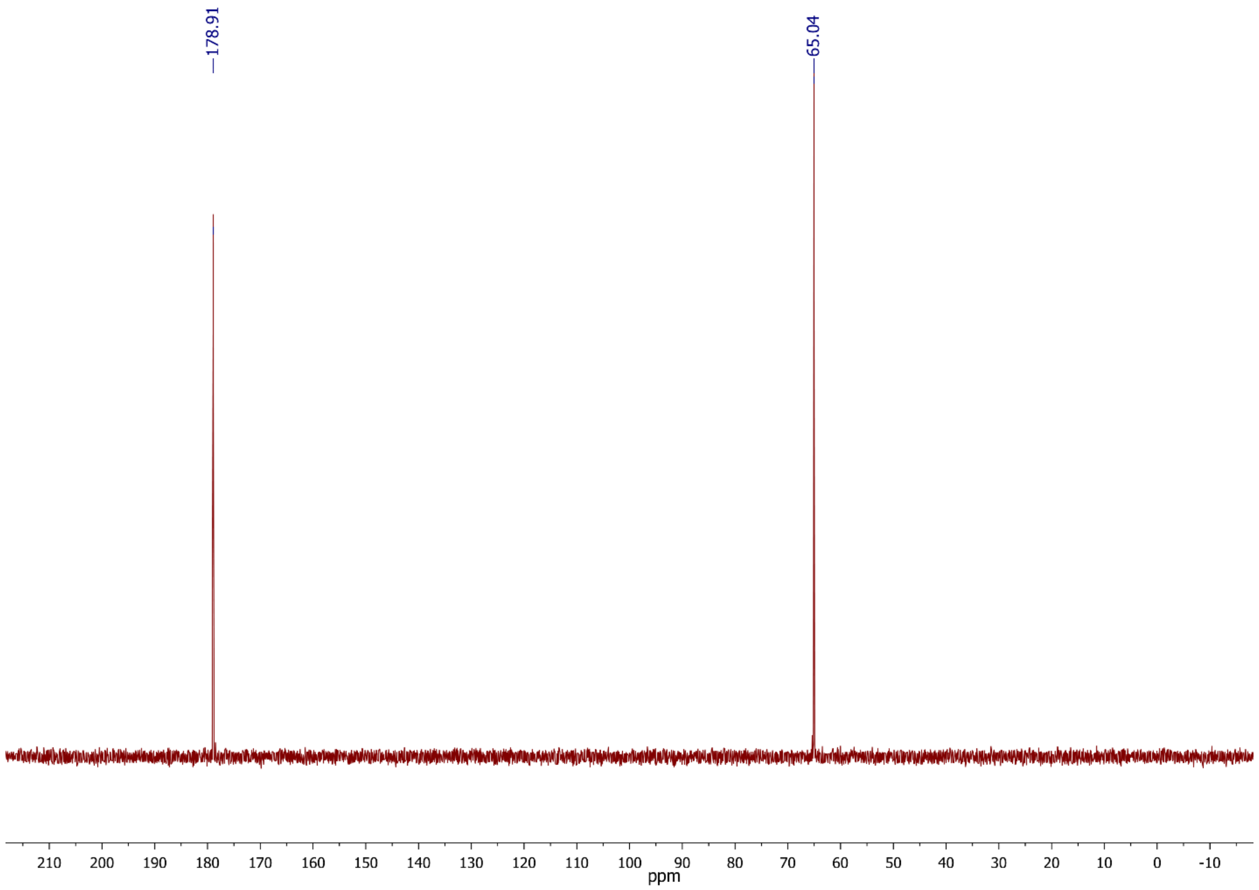

Figure S2. ^{13}C NMR spectrum of crystal solution $Na_2[Hf(NTA)_2]\cdot 3H_2O$ (1) in D_2O at 298K

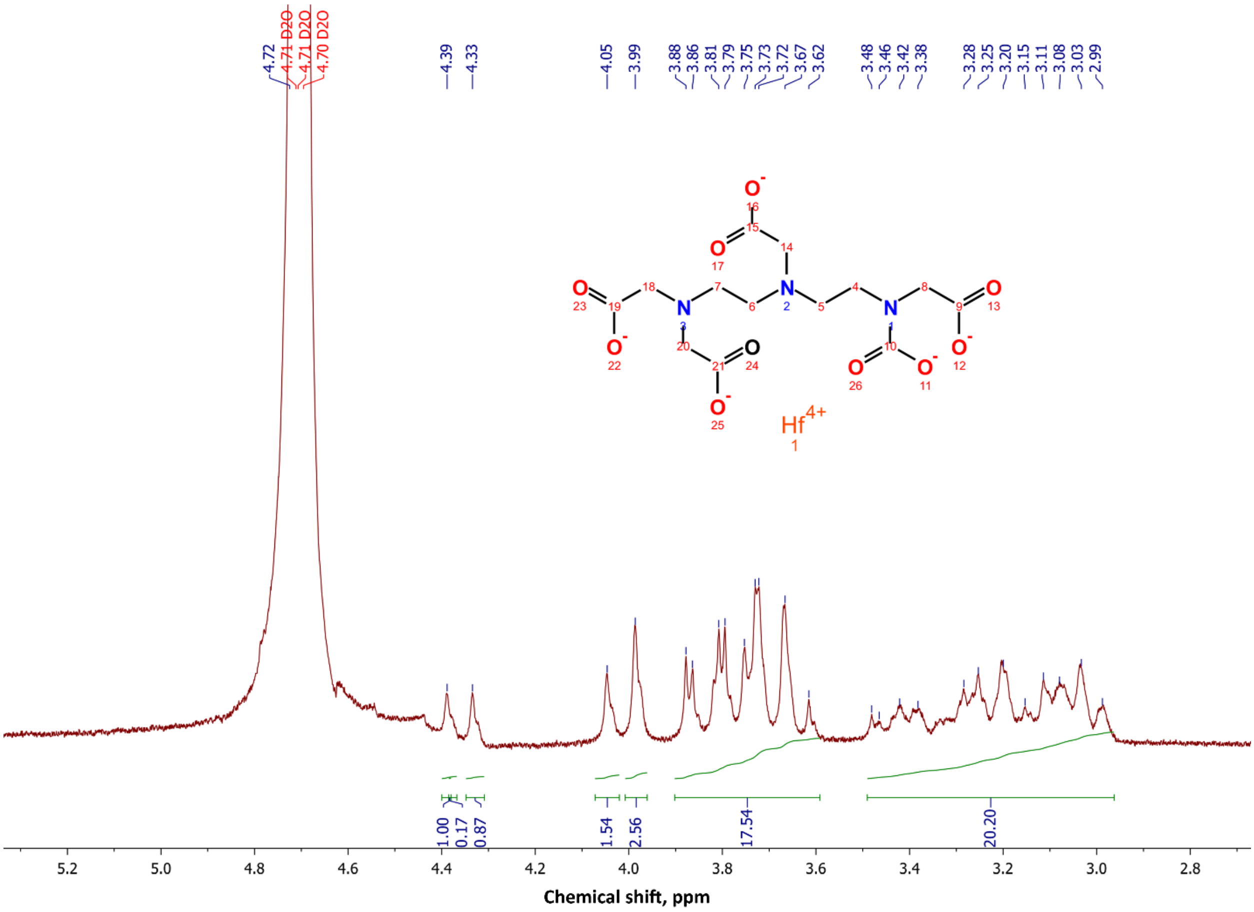

Figure S3. ^1H NMR spectrum of crystal solution Na[HfDTPA]·3H_2O (**2**) in D_2O at 298K

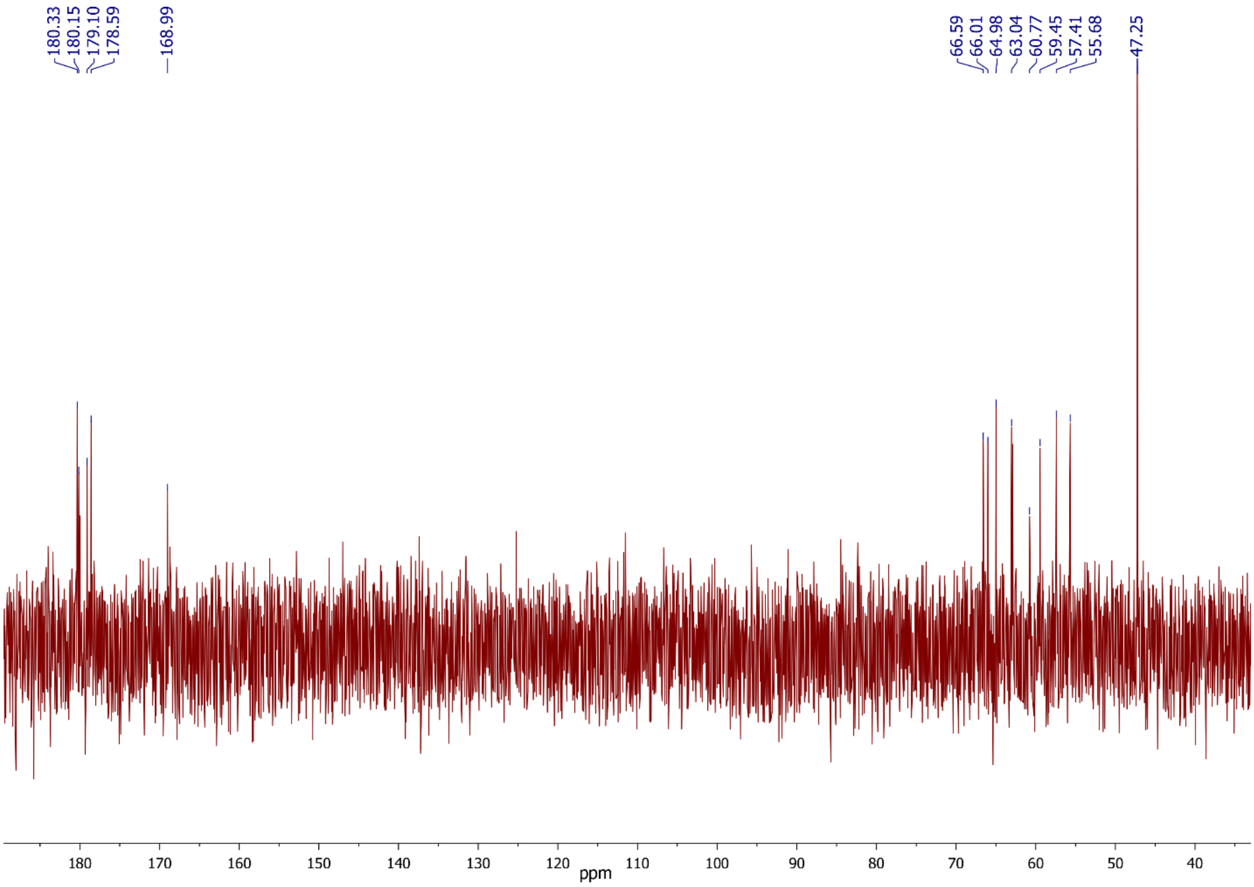

Figure S4. ^{13}C NMR spectrum of crystal solution $Na[HfDTPA]\cdot 3H_2O$ (1) in D_2O at 298K

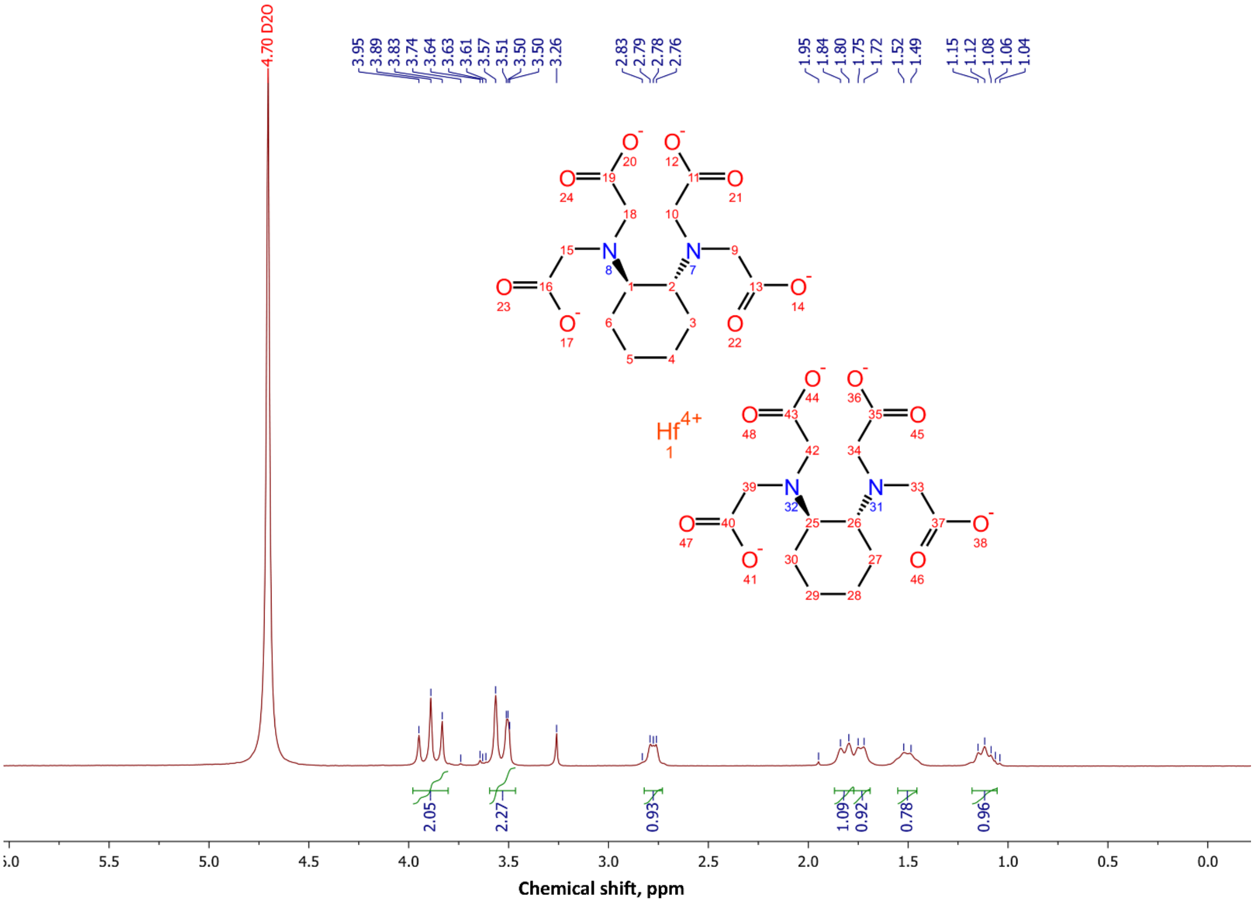

Figure S5. ^{1}H NMR spectrum of crystal solution [HfCDTA(H_2O)$_2$] (**3**) in D_2O at 298K

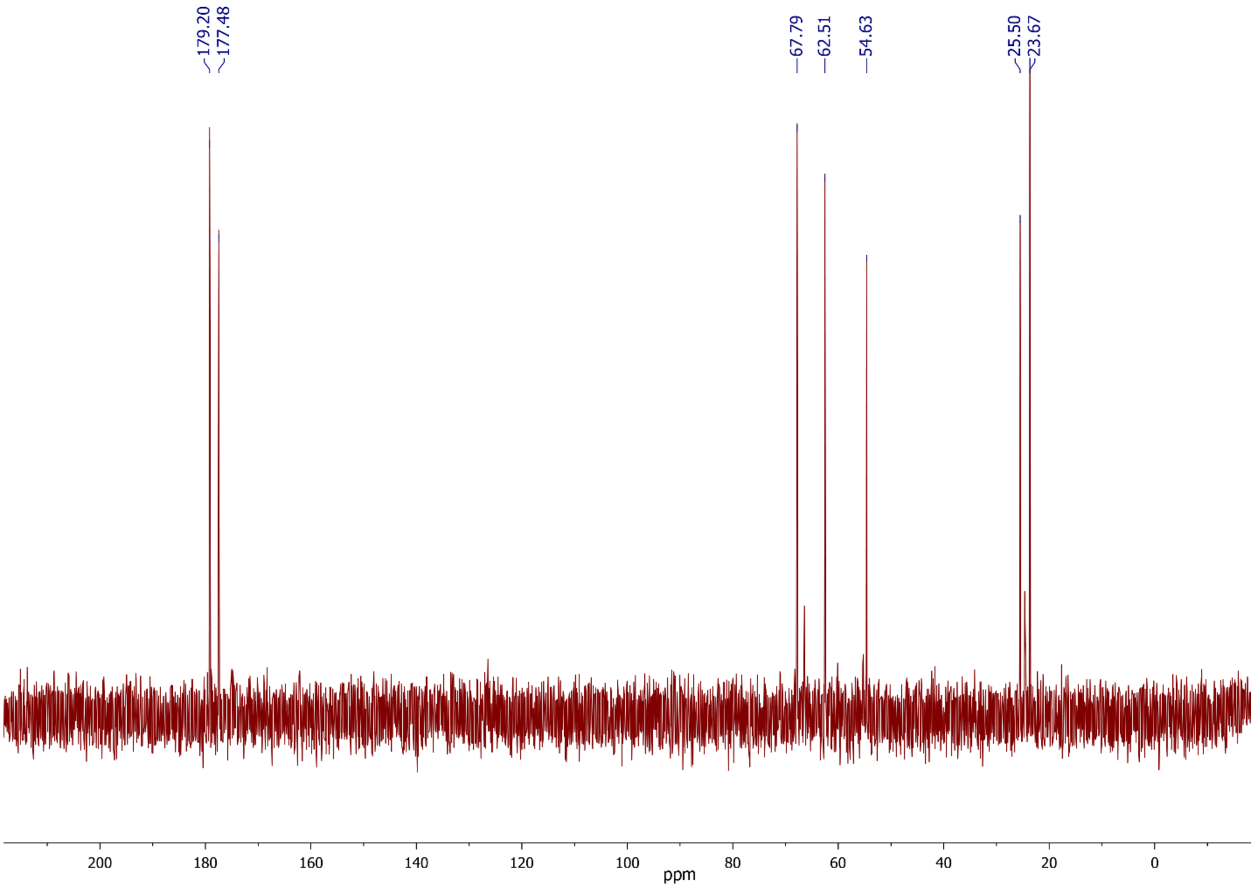

Figure S6. ^{13}C NMR spectrum of crystal solution [HfCDTA$(H_2O)_2$] (1) in D_2O at 298K

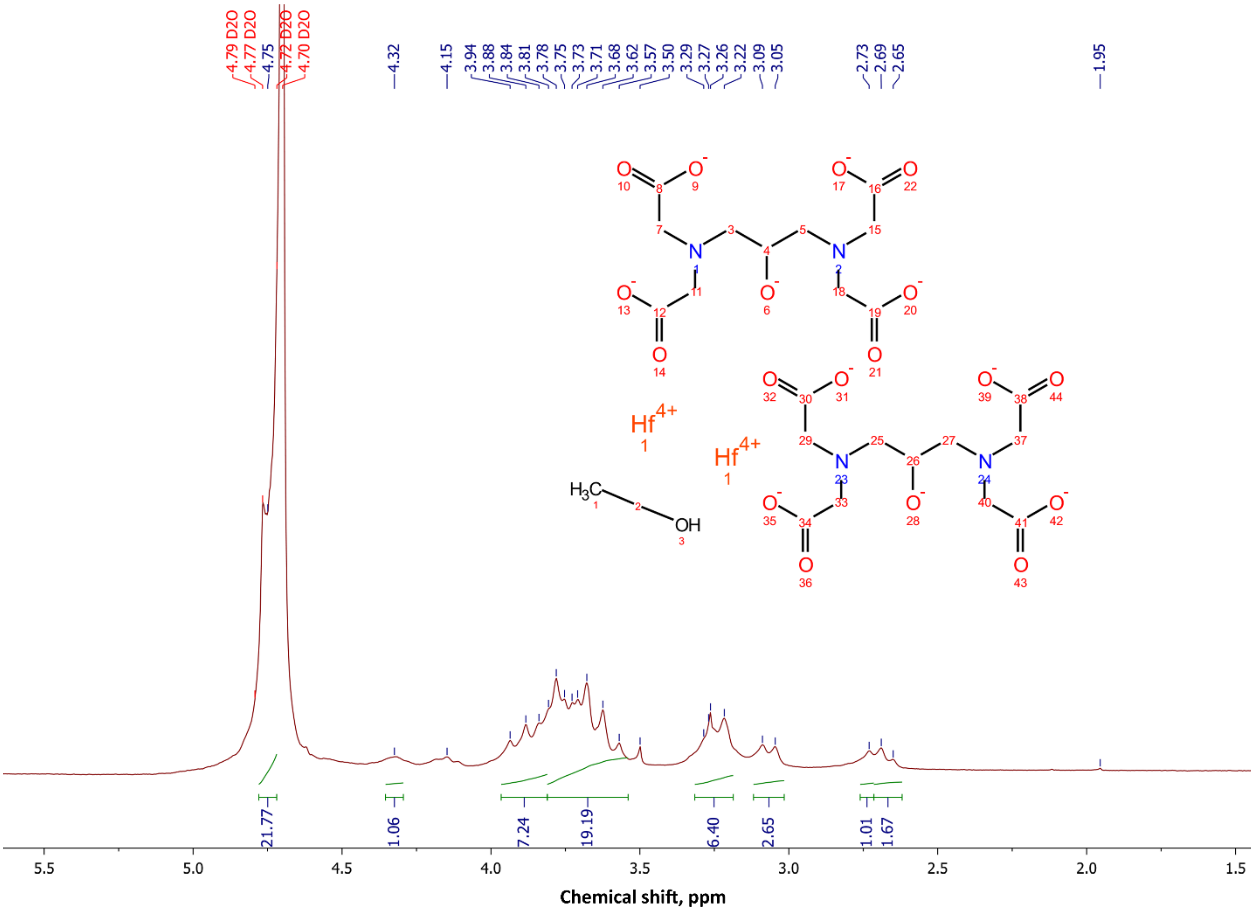

Figure S7. ^{1}H NMR spectrum of crystal solution $Na[Hf_2(dpta)_2]\cdot 7.5H_2O\cdot 0.5C_2H_5OH$ (**4**) in D_2O at 298K

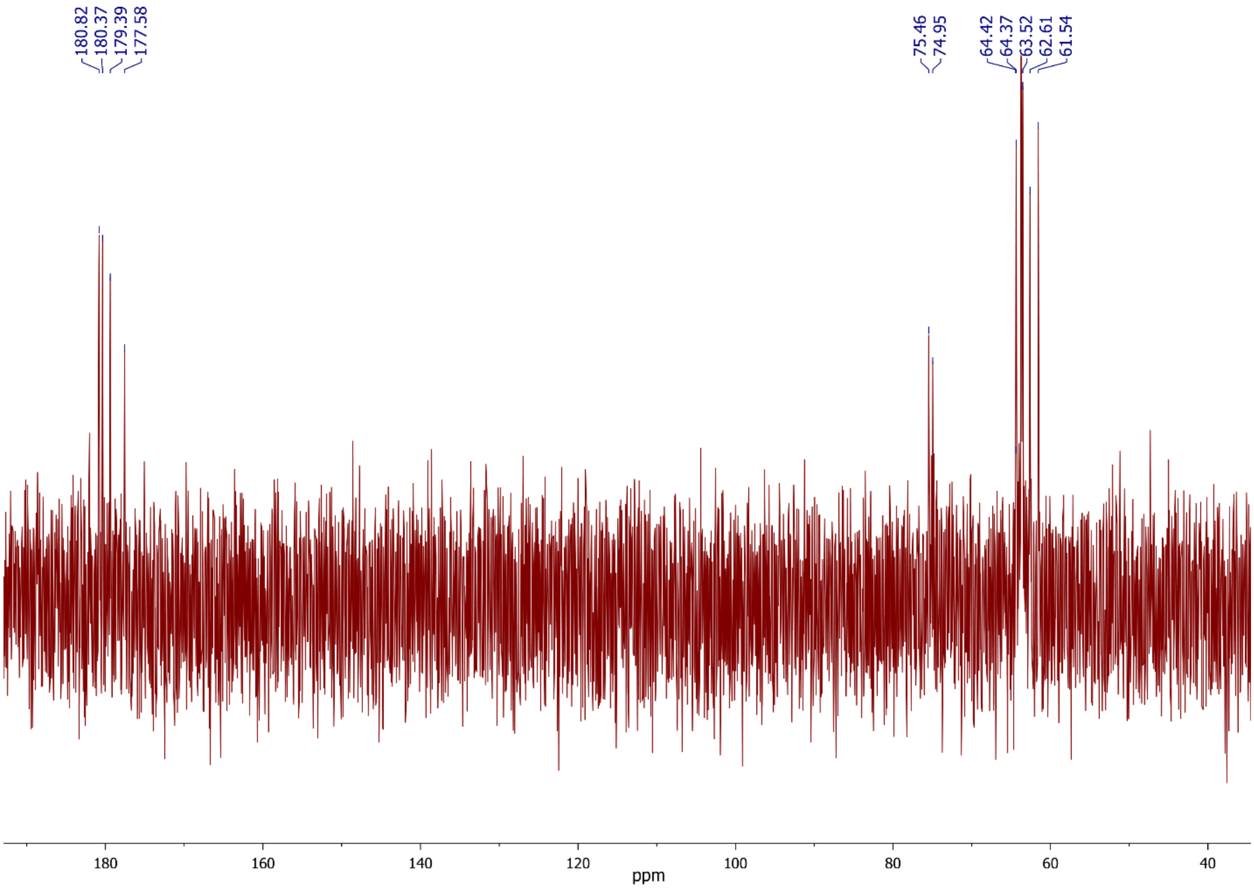

Figure S8. ^{13}C NMR spectrum of crystal solution $Na[Hf_2(dpta)_2]\cdot7.5H_2O\cdot0.5C_2H_5OH$ (1) in D_2O at 298K

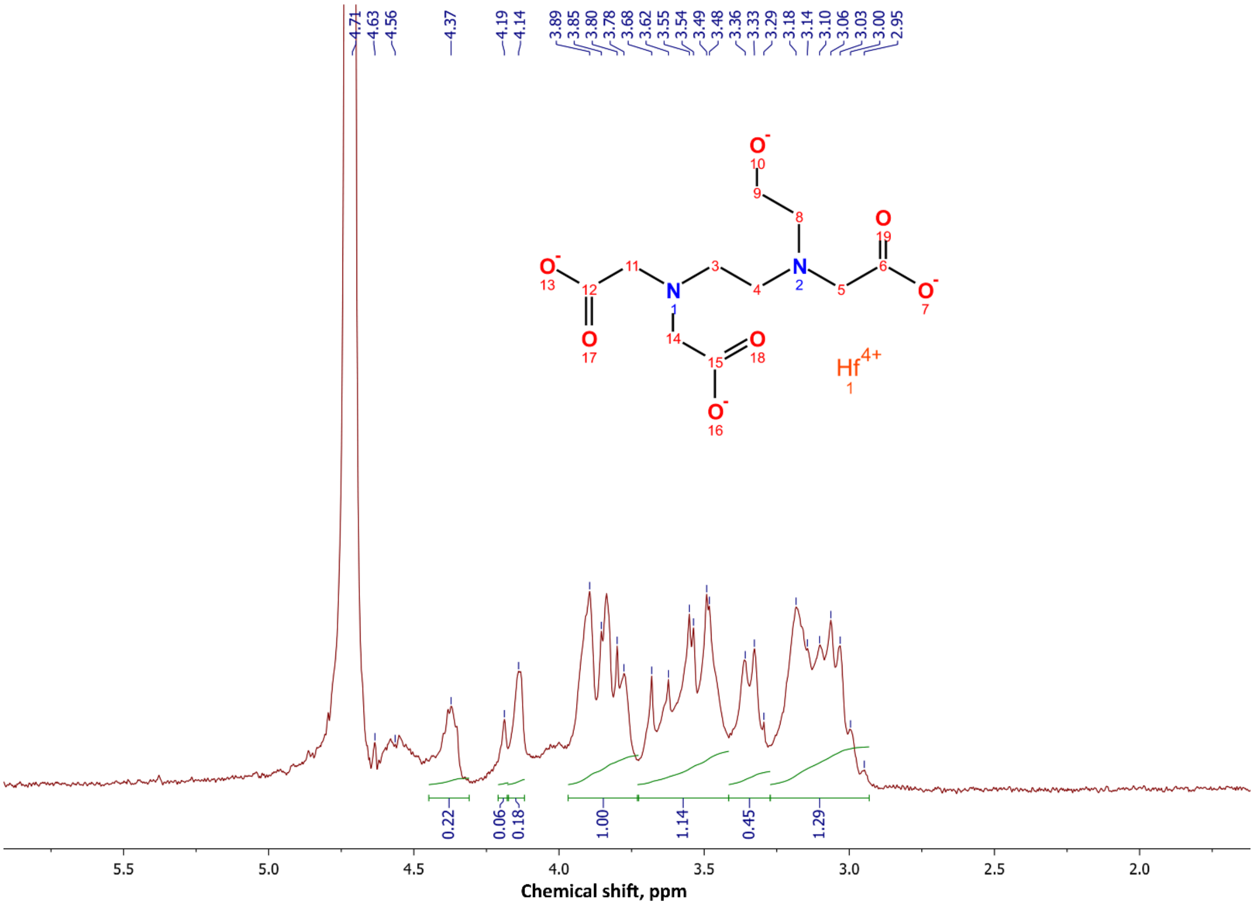

Figure 9. ^{1}H NMR spectrum of solution $HfCl_4$ and Na3HEDTA (**5**).in D_2O at 298K

3. X-ray diffraction data

Table S1. Crystallographic data and experimental and refinement details for compounds (I–III).

Identification code	20kub75_0m_a (**I**)	20kub29_0m_a (**II**)	20kub92 (**III**)
Empirical formula	$C_{12}H_{20}HfN_2Na_2O_{16}$	$C_{14}H_{24}HfN_3NaO_{13}$	$C_{14}H_{22}HfN_2O_{10}$
Formula weight	672.77	643.84	556.82
Temperature/K	100	150	296.15
Crystal system	monoclinic	monoclinic	orthorhombic
Space group	C2/m	$P2_1/c$	Pnma
a/Å	19.6130(5)	9.6673(3)	13.8611(5)
b/Å	10.9862(2)	13.1485(4)	10.2579(4)
c/Å	10.6323(2)	15.2870(4)	12.5307(4)
α/°	90	90	90
β/°	119.3290(10)	90.7260(10)	90
γ/°	90	90	90
Volume/Å^3	1997.31(7)	1942.98(10)	1781.69(11)
Z	4	4	4
ρ_{calc}g/cm^3	2.237	2.201	2.076
μ/mm^{-1}	5.356	5.471	5.913
F(000)	1312.0	1264.0	1088.0
Crystal size/mm^3	0.3 × 0.25 × 0.15	0.5 × 0.5 × 0.03	0.5 × 0.4 × 0.25
Radiation	MoKα (λ = 0.71073)	MoKα (λ = 0.71073)	MoKα (λ = 0.71073)
2Θ range for data collection/°	4.394 to 54.998	4.214 to 53.994	5.132 to 51.996
Index ranges	-22 ≤ h ≤ 25, -13 ≤ k ≤ 14, -13 ≤ l ≤ 12	-12 ≤ h ≤ 12, -16 ≤ k ≤ 16, -19 ≤ l ≤ 19	-11 ≤ h ≤ 17, -12 ≤ k ≤ 8, -13 ≤ l ≤ 15
Reflections collected	9411	18847	6215
Independent reflections	2409 [R_{int} = 0.0241, R_{sigma} = 0.0227]	4235 [R_{int} = 0.0517, R_{sigma} = 0.0439]	1840 [R_{int} = 0.0376, R_{sigma} = 0.0378]
Data/restraints/parameters	2409/0/252	4235/0/303	1840/7/193
Goodness-of-fit on F^2	1.129	1.079	1.358
Final R indexes [I>=2σ (I)]	R_1 = 0.0206, wR_2 = 0.0482	R_1 = 0.0286, wR_2 = 0.0632	R_1 = 0.0407, wR_2 = 0.1056
Final R indexes [all data]	R_1 = 0.0213, wR_2 = 0.0485	R_1 = 0.0328, wR_2 = 0.0648	R_1 = 0.0424, wR_2 = 0.1063

Table S2. Crystallographic data and experimental and refinement details for compounds (IV).

Identification code	20kub80_0m_a (**IV**)
Empirical formula	$C_{23}H_{41.9}Hf_2N_4Na_2O_{26}$
Formula weight	1193.46
Temperature/K	100
Crystal system	triclinic
Space group	P-1
a/Å	9.3901(2)
b/Å	14.0010(3)
c/Å	14.9797(4)
α/°	75.9640(10)
β/°	86.3100(10)
γ/°	73.5280(10)
Volume/Å^3	1832.16(7)
Z	2
ρ_{calc}g/cm^3	2.163
μ/mm^{-1}	5.791
F(000)	1164.0
Crystal size/mm^3	0.3 × 0.3 × 0.02
Radiation	MoKα (λ = 0.71073)
2Θ range for data collection/°	5.126 to 54.998
Index ranges	-11 ≤ h ≤ 12, -17 ≤ k ≤ 18, -19 ≤ l ≤ 19
Reflections collected	19715
Independent reflections	8387 [R_{int} = 0.0342, R_{sigma} = 0.0485]
Data/restraints/parameters	8387/6/510
Goodness-of-fit on F^2	1.053

Reference

1. G.M. Sheldrick. Crystal structure refinement with SHELXL. *Acta Cryst* **2015**, *71*, 3–8
2. Dolomanov, O.V., Bourhis, L.J., Gildea, R.J., Howard, J.A.K. and Puschmann, H. OLEX2: A Complete Structure Solution, Refinement and Analysis Program. *Appl. Cryst*. **2009**, *42*, 339-341.
3. M.J. Turner, J.J. McKinnon, S.K. Wolff, D.J. Grimwood, P.R. Spackman, D. Jayatilaka M.A.S. CrystalExplorer17.5. Perth, Australia: University of Western Australia. 2017.
